# MCM ring hexamerization is a prerequisite for DNA-binding

**DOI:** 10.1093/nar/gkv914

**Published:** 2015-09-13

**Authors:** Clifford A. Froelich, Amanda Nourse, Eric J. Enemark

**Affiliations:** 1 Department of Structural Biology, St Jude Children's Research Hospital, 262 Danny Thomas Place, Mail Stop 311, Memphis, TN 38105, USA; 2 Molecular Interaction Analysis Shared Resource, St Jude Children's Research Hospital, 262 Danny Thomas Place, Mail Stop 311, Memphis, TN 38105, USA

## Abstract

The hexameric Minichromosome Maintenance (MCM) protein complex forms a ring that unwinds DNA at the replication fork in eukaryotes and archaea. Our recent crystal structure of an archaeal MCM N-terminal domain bound to single-stranded DNA (ssDNA) revealed ssDNA associating across tight subunit interfaces but not at the loose interfaces, indicating that DNA-binding is governed not only by the DNA-binding residues of the subunits (MCM ssDNA-binding motif, MSSB) but also by the relative orientation of the subunits. We now extend these findings by showing that DNA-binding by the MCM N-terminal domain of the archaeal organism *Pyrococcus furiosus* occurs specifically in the hexameric oligomeric form. We show that mutants defective for hexamerization are defective in binding ssDNA despite retaining all the residues observed to interact with ssDNA in the crystal structure. One mutation that exhibits severely defective hexamerization and ssDNA-binding is at a conserved phenylalanine that aligns with the mouse *Mcm4(Chaos3)* mutation associated with chromosomal instability, cancer, and decreased intersubunit association.

## INTRODUCTION

The Minichromosome Maintenance (MCM) complex forms an essential hexameric-ring helicase that unwinds DNA at the replication fork (1–3). In eukaryotes, the MCM ring consists of six different homologous proteins (Mcm2–7) (1,2,4,5). In the case of several archaeal organisms, a single MCM protein forms a hexameric ring that can unwind duplex DNA *in vitro* (6–12). Electron microscopy studies show that these MCM complexes form a two-tiered hexameric ring structure (6,13–22). These archaeal MCM homohexamers retain essential core MCM activities and therefore represent simplified versions of eukaryotic Mcm2–7 heterohexamers, and on this basis have served as powerful models for investigating essential features of MCM helicase structure and function. MCM proteins have three conserved domains: an N-terminal domain (MCM_N_) that mediates the head-to-head interaction of the initial double-hexamer (23); a conserved AAA+ ATPase domain (12,24–27); and a short helix-turn-helix domain at the C-terminus (28–30). Crystal structures of the N-terminal domains of *Methanobacterium thermautotrophicus* (*Mt*MCM_N_) (23), *Sulfolobus solfataricus* (*Sso*MCM_N_) (31) and *Pyrococcus furiosis* (*Pf*MCM_N_) (32) show a consistent hexameric ring structure with three subdomains (23): a helical subdomain-A, a Zn-binding subdomain-B and an OB-fold subdomain-C.

The N-terminal domain OB-fold subdomain-C is essential for ring hexamerization (23,33) and also contains several residues critical for binding DNA (8,9,23,32,34–36). The crystal structure of *Pf*MCM_N_ bound to single-stranded DNA (ssDNA) (32) revealed that subdomain-C binds ssDNA in the plane of the hexameric ring via conserved residues defined as the MCM single-stranded DNA-binding motif (MSSB) (32). The identified interactions could play a role during initial loading of the MCM helicase in the vicinity of replication origins, during activation of the helicase, or during the processive DNA unwinding that follows helicase activation. Although the *Pf*MCM_N_ hexamer consists of six identical subunits that inherently have six identical MSSB amino acid sequences, DNA-binding was not equivalent at each subunit. Instead, the presence of ssDNA was associated with more closely spaced subunit interfaces. Specifically, ssDNA was observed when the intersubunit spacing (defined by the distance between the R201 Cα atom of one subunit and the E127 Cα atom of the neighboring subunit) was less than 7.5 Å, and ssDNA was not observed when this distance exceeded 8.4 Å. This correlation indicates that the relative position and orientation of subunits are strong determinants of DNA-binding and hence that the oligomerization and DNA-binding activities of subdomain-C are coupled.

Based on the coupling of these activities, MCM mutations that affect intersubunit association could significantly impair binding to DNA. The *Mcm4(Chaos3)* mutation identified in mice (*Mm*) causes a point mutation of a conserved phenylalanine in Mcm4 (F345I) (37,38), and female mice homozygous for this mutation often develop mammary adenocarcinoma (37,38). The *Mm*Mcm4 F345 residue is located in subdomain-C of the N-terminal domain (38), and the F345I mutation severely disrupts interaction of Mcm4 with the neighboring Mcm6 subunit (39). The key role that this conserved phenylalanine plays in the association of Mcm4 and Mcm6 suggests it could provide a useful tool to investigate potential correlation between MCM oligomerization and binding to DNA.

In this paper, we investigate the role of MCM_N_ intersubunit interactions on ssDNA-binding. We show that wild-type *Pf*MCM_N_ exists in multiple oligomeric forms in solution, but that ssDNA exclusively associates with hexameric *Pf*MCM_N_. Hexamerization-defective mutants show severe defects in binding ssDNA. All of the defective mutants have an intact MSSB, and thus retain the residues needed to bind ssDNA. We show that mutation of the *Pf*MCM_N_ residue analogous to that of the *MmMcm4(Chaos3)* mutation is severely defective for oligomerization and binding to ssDNA. We also identify a mutant that forms a pentameric ring in solution and in the crystal structure, and we show that this mutant does not bind ssDNA. Collectively, our results further support a role for specifically oriented subunits in order for the MCM_N_ hexamer to bind ssDNA.

## MATERIALS AND METHODS

### Cloning, mutagenesis, expression and purification

The expression construct for a SUMO-fusion (the SUMO fusion cloning vector was the generous gift of Dr Christopher D. Lima) (40) of the amino-terminal domain of *Pyrococcus furiosis* MCM, *Pf*MCM_N_-WT, has been reported previously (32). The other expression constructs for this study were prepared by site-directed mutagenesis: *Pf*MCM_N_-ΔZFD (deletion of residues 131–181, pCF045.3), *Pf*MCM_N_-F179A (pCF052.1) and *Pf*MCM_N_-βT (K233A/R234A/K236A; pCF076.1). The coding region of each construct was verified by DNA sequencing. Proteins were recombinantly expressed and purified as described previously (32), including removal of the SUMO-tag by Ulp1 protease (the Ulp1 protease plasmid was the generous gift of Dr Christopher D. Lima) (40). In the final size-exclusion chromatography step of purification, *Pf*MCM_N_-ΔZFD and -F179A elute at a volume similar to *Pf*MCM_N_-WT, consistent with a monomer. In contrast, *Pf*MCM_N_-βT elutes at an earlier volume from the size-exclusion column at a position most consistent with a trimer, suggesting that a larger oligomer of *Pf*MCM_N_-βT is more stable than in the case of *Pf*MCM_N_-WT. The identities and masses of all proteins were verified by SDS-PAGE, MALDI-TOF–TOF and LC–MS.

### Analytical ultracentrifugation (AUC)

Experiments were conducted in a ProteomeLab XL-I analytical ultracentrifuge (Beckman Coulter, Indianapolis, IN, USA) following standard protocols (41) unless stated otherwise. All samples in a buffer containing 20 mM HEPES, pH 7.6, 200 mM NaCl, 5 mM β-mercaptoethanol were loaded into double sector charcoal-filled centerpieces with 12 mm path lengths and either quartz or sapphire windows. The density and viscosity of the ultracentrifugation buffer were calculated using the software SEDNTERP (provided by J. Philo) (42). The partial specific volumes and the molecular masses of the proteins, *Pf*MCM_N_-WT, -ΔZFD, -F179A and -βT were calculated based on their amino acid compositions in SEDFIT (https://sedfitsedphat.nibib.nih.gov/software/default.aspx). The molecular mass of Flc-T40 (5′-fluorescein-tagged, poly-deoxythymidine of 40 nucleotide length, 12 922 Da) presuming a partial specific volume of 0.55 ml/g, was converted to an adjusted molecular mass of 22 015 Da using the partial specific volume of 0.73 g/ml, the value for the proteins at 4°C, Supplementary Table S1.

For the sedimentation velocity experiments, the cell assemblies, containing identical sample and reference buffer volumes of 360–400 μl, were placed in a rotor and allowed to equilibrate to 20°C at rest before accelerating to 50 000 rpm. Both Rayleigh interferometric fringe displacement data and absorbance optical data at 280 or 490 nm (for the samples containing Flc-T40) were collected continuously for 10 h.

The sedimentation velocity data were modeled with diffusion-deconvoluted sedimentation coefficient distributions *c*(*s*) in SEDFIT (43) using algebraic noise decomposition and with signal-average frictional ratio and meniscus position refined with non-linear regression. The *s*-values were corrected for time (44) and finite acceleration of the rotor was accounted for in the evaluation of Lamm equation solutions (45). Maximum entropy regularization was applied at a confidence level of *P* = 0.68. Isotherm data of the signal-average *s*-values, *s*_w_, of the total sedimenting system derived from integration of the complete *c*(*s*) distributions at various concentrations of all *Pf*MCM_N_-WT AUC sedimentation velocity data were fitted to a reversible monomer-hexamer self-association system using SEDPHAT (https://sedfitsedphat.nibib.nih.gov/software/default.aspx) (46).

Sedimentation equilibrium experiments were performed following the protocol described in (41). Sedimentation equilibrium for apoproteins were attained at a rotor temperature of 20°C and increasing rotor speeds of 15 000 rpm (24 h), 20 000 rpm (24 h) and 25 000 rpm (24 h) for *Pf*MCM_N_-WT and 10 000 rpm (38 h) 18 000 rpm (26 h) and 25 000 rpm (18 h) for *Pf*MCM_N_-βT. Protein at concentrations between 12 and 51 μM (130 μl) were loaded into double-sector centerpieces and absorbance data acquired at 280 nm in 0.001 cm radial increments with 20 replicates for each point. Global least squares modelling were performed at multiple rotor speeds with the software SEDPHAT (https://sedfitsedphat.nibib.nih.gov/software/default.aspx) using the reversible monomer-hexamer (1↔6) or monomer-pentamer (1↔5) self-association model (47). The protein:Flc-T40 (83 μM:15.2 μM) mixtures were diluted while keeping the concentration ratio of 0.9 constant, and sedimentation equilibrium were attained at a rotor temperature of 4°C at increasing speeds of 12 000 rpm (57 h), 21 000 rpm (34 h) and 35 000 rpm (12 h) for *Pf*MCM_N_-WT and 12 000 rpm (51 h), 21 000 rpm (41 h) and 35 000 rpm (20 h) for *Pf*MCM_N_-βT. Absorbance data were collected at 490 nm in 0.001 cm radial increments with 20 replicates for each point. The molar extinction coefficient of the Flc-T40 at 490 nm, 66 836 absorbance units/(M·cm), was calculated from the measured absorbance at 490 nm of pure Flc-T40 using its absorbance at 260 nm and molar extinction coefficient at 260 nm (346 400 absorbance units/(M·cm)) as a measure of concentration. Global multispeed analysis of the sedimentation equilibrium absorbance data was carried out in SEDPHAT using the reversible single site hetero-association model (A+B ↔ AB) with A the free Flc-T40 (monomer) species and B the hexamer-protein species (47,48), Supplementary Table S2.

### Crystallization, data collection, structure solution and refinement

Diffraction data were collected at SER-CAT beam lines 22-ID (*Pf*MCM_N_-ΔZFD and *Pf*MCM_N_-F179A) and 22-BM (*Pf*MCM_N_-βT) at the Advanced Photon Source at Argonne National Lab at 1.0 Å wavelength, 100 K. Data were integrated and scaled with the HKL-2000 package (49). Figures were prepared with variety of software (50–52). Statistics for the crystal structures are provided in Supplementary Table S3.

Crystals of *Pf*MCM_N_-F179A were grown by hanging drop vapor diffusion in a ratio of 1:1 (protein:well solution). The stock protein solution was 340 μM with the well solution consisting of 50 mM sodium cacodylate, pH 6.5, 1 mM spermine, 35 mM MgCl_2_, 2.45 M ammonium sulfate. Crystals were cryoprotected in a 2.1 M lithium sulfate/well solution (2:1) and flash frozen. Data were collected in 0.25° oscillations for a total of 180° of crystal rotation and were integrated and scaled to 3.20 Å resolution. Phaser (53) placed 10 copies of a monomer of *Pf*MCM_N_-WT (32) as a central pentameric ring with five exterior monomers. The model was refined with CNS (54,55) and with Refmac5 (56). Following coordinate and group B-factor refinement with CNS (54,55), the final coordinate refinement was carried out with Refmac5 (56). A Ramachandran plot calculated by Procheck (57) indicated the following statistics: core: 2040 (90.3%); allowed: 204 (9.0%); generously allowed: 8 (0.4%); disallowed: 8 (0.4%). Each of the eight disallowed residues is one of the copies of D209, which resides at the end of a tight structural turn of an ‘Allosteric Communication Loop’ (ACL) (36,58). The D209 phi/psi angles are just outside the allowed region of the plot (chain A-J phi/psi = 55.1/−116.2; 57.1/−116.0; 56.2/−115.6; 55.0/−116.9; 56.1/−115.2; 51.1/−116.8; 52.4/−120.8; 52.4/−117.5; 50.1/−121.4; 55.1/−117.5), very close to the angles observed, in the allowed region, for the wild-type hexamer (PDB 4POF chain A-F phi/psi = 53.1/−121.6; 55.1/−124.4; 52.5/−123.1; 49.8/−122.9; 49.4/−123.9; 47.4/−126.1) (32).

Crystals of *Pf*MCM_N_-βT were grown by hanging drop vapor diffusion. The stock protein (340 μM) was mixed with the well solution (100 mM Bis–Tris, pH 5.5, 1.75 M ammonium sulfate) in a ratio of 2:1. Crystals were cryoprotected in a solution composed of one-third well solution and two-thirds 2.5 M ammonium sulfate, 25% glycerol solution and flash frozen. Data were collected in 0.5° oscillations for a total of 180° of crystal rotation. Data were integrated and scaled to 3.20 Å resolution. The *Pf*MCM_N_-βT dataset was placed in the same setting and assigned the same test set as the roughly isomorphic *Pf*MCM_N_-F179A dataset. Phaser (53) placed 10 copies of a monomer of *Pf*MCM_N_-WT (32) as a central pentameric ring with five exterior monomers. The model was refined with CNS (54,55) and with Refmac5 (56). Following coordinate and group B-factor refinement with CNS (54,55), the final coordinate refinement was carried out with Refmac5 (56). A Ramachandran plot calculated by Procheck (57) indicated the following statistics: core: 2033 (89.8%); allowed: 222 (9.8%); generously allowed: 0 (0%); disallowed: 10 (0.4%). The 10 disallowed residues consist of the 10 individual copies of residue D209. This residue is at the end of a tight structural turn of the ‘Allosteric Communication Loop’ (ACL) (36,58) with phi/psi angles placing it at the border of the allowed and disallowed regions of the plot (chain A-J phi/psi = 54.6/−116.3; 53.8/−114.9; 54.3/−115.3; 54.4/−115.0; 57.2/−112.9; 42.0/−116.9; 39.8/−118.8; 43.7/−118.2; 41.4/−117.5; 45.0/−117.9)—very close to the angles observed, in the allowed region, for the wild-type hexamer (PDB 4POF chain A-F phi/psi = 53.1/−121.6; 55.1/−124.4; 52.5/−123.1; 49.8/−122.9; 49.4/−123.9; 47.4/−126.1) (32).

Crystals of *Pf*MCM_N_-ΔZFD were grown by hanging drop vapor diffusion. The stock protein (425 μM) was mixed with well solution (50 mM ammonium fluoride, 21% PEG 3350) in a ratio of 1:2, respectively. Crystals were cryoprotected in 25% ethylene glycol and flash frozen. Data were collected in 0.5° oscillations for a total of 180° of crystal rotation and were integrated and scaled to 1.55 Å resolution. Phaser (53) placed two copies of a monomer of *Pf*MCM_N_-WT with the Zn-binding domain removed. The model was refined with CNS (54,55) and with Refmac5 (56). The final refinement was carried out with Refmac5 (56) with individual atomic B-factors. A Ramachandran plot calculated by Procheck (57) indicated the following statistics: core: 302 (93.8%); allowed: 20 (6.2%); generously allowed: 0 (0%); disallowed: 0 (0%).

### Electromobility shift assay (EMSA)

DNA-binding assays were performed as previously reported (32). Briefly, Flc-T40 ssDNA (200 nM) (Sigma-Aldrich, St. Louis, MO, USA) was allowed to bind for 1 h at 25°C. Protein concentration was titrated, and the species were resolved by 4–20% gradient 1X TBE PAGE. EMSAs were visualized using a GelDoc system (Fuji LAS-4000, GE Healthcare, Piscataway, NJ, USA) with a SYBR-Green filter exposed for 8 s. For mixtures of wild-type and mutant proteins, individually purified proteins were first isolated as monomers by size-exclusion chromatography and then combined in the desired ratios prior to concentration. These solutions were concentrated and used in EMSA experiments similar to above.

## RESULTS

### Concentration-dependent *Pf*MCM_N_-WT hexamer

**Figure 1.**
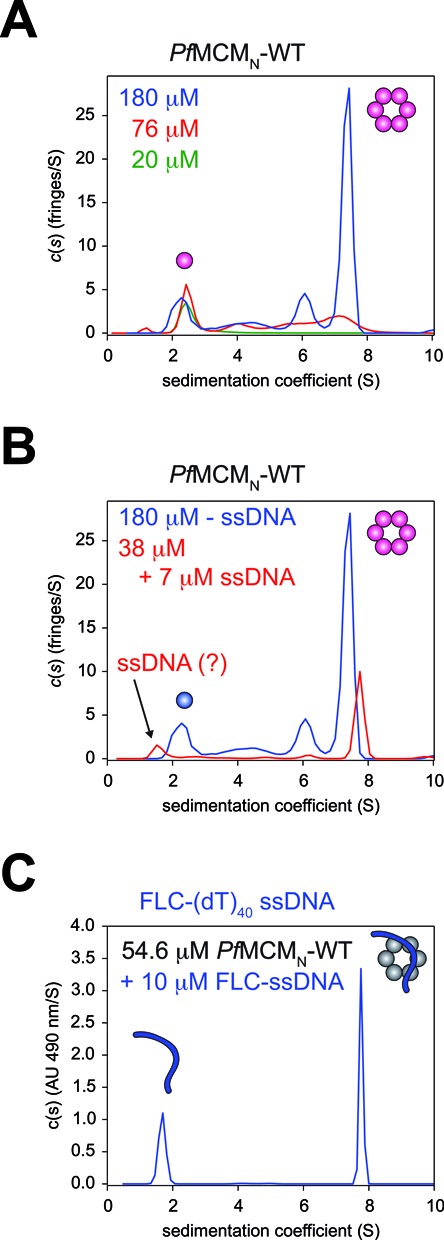
Oligomerization of *Pf*MCM_N_-WT. (**A**) AUC sedimentation coefficient distribution of *Pf*MCM_N_-WT shows two stable species consistent with a monomer and a hexamer. The distribution between these species is protein concentration-dependent. (**B**) Addition of 40-mer oligo-dT ssDNA stabilizes the hexameric form of *Pf*MCM_N_-WT. (**C**) AUC sedimentation coefficient distribution when monitoring fluorescein absorbance at 490 nm of 5′-fluorescein-40-mer oligo-dT (Flc-T40) in the presence of *Pf*MCM_N_-WT shows two peaks: unbound Flc-T40 (*s*_20_ = 1.67 S) and Flc-T40 bound to hexameric *Pf*MCM_N_-WT (*s*_20_ = 7.77 S). The *Pf*MCM_N_-WT concentration is 54.5 μM and the FLC-T40 concentration is 10 μM.

*Pf*MCM_N_-WT purifies as a monomer but forms a hexameric ring in crystal structures (32), suggesting that hexamerization is protein concentration-dependent. To investigate the relationship between protein concentration and hexamer formation, we performed AUC sedimentation velocity and equilibrium experiments with varying concentrations of *Pf*MCM_N_-WT. Samples were initially concentrated to 510 μM (15 mg/ml) and then diluted in equivalent buffer solution. At the highest *Pf*MCM_N_-WT concentration (350 μM), a single predominant species is observed in the sedimentation velocity profile. Co-interpretation of the sedimentation velocity and equilibrium data suggests that this species is an elongated oligomer, larger than a single hexamer (described below). While the specific structure of this oligomer is not uniquely determined by these experiments, the data are consistent with a 12 subunit double-hexamer analogous to that observed crystallographically for *Mt*MCM_N_ (23), Supplementary Table S1. At lower concentrations of *Pf*MCM_N_-WT of 180 μM and 76 μM, sedimentation peaks for two predominant stable species are observed that are consistent with monomeric and hexameric species (Figure 1A; Supplementary Table S1). The percentage of protein in the hexameric fraction decreases as the protein is diluted, from 60% (180 μM) to 37% (76 μM) (Supplementary Table S1), consistent with protein-concentration-dependent hexamerization. To quantify the stability of the *Pf*MCM_N_-WT hexamer, the sedimentation equilibrium experiments were analyzed with a A_6_ ↔ 6A model (where A_6_ is hexamer and A is monomer) to determine the protein concentration where half of the total protein is monomeric and half is hexameric, *K*_d1–6_ = 60 μM (Supplementary Table S2). This value is consistent with the 58 μM value obtained independently by fitting the isotherm of signal-average s-values from multiple sedimentation velocity experiments obtained via interference optics at various concentrations (Supplementary Figure S1).

### DNA stabilizes the *Pf*MCM_N_-WT hexamer

We next determined the role of DNA in stabilizing the concentration-dependent hexamer of *Pf*MCM_N_-WT. Using a concentration of *Pf*MCM_N_-WT (38 μM) expected to yield a heterogeneous profile with significant monomeric species (Figure 1A, Supplementary Table S1), we added ssDNA (40-mer oligo-dT, T40) and performed AUC experiments using interference optics. The sedimentation velocity profile shows the percentage of *Pf*MCM_N_-WT hexamer at this dilution is larger when T40 is present (with T40: 72% hexamer at 38 μM versus without T40: 60% hexamer at 180 μM and 37% hexamer at 76 μM); Supplementary Table S1, Figure 1A and B). Dilution of the DNA-containing sample yields decreased percentages of hexamer, from 82% (128 μM) to 72% (45 μM) and 55% (15 μM), indicating that the DNA-promoted hexamer is also concentration-dependent (Supplementary Table S1, Supplementary Figure S2).

### *Pf*MCM_N_-WT MCM cooperatively binds ssDNA as a hexamer

To determine the distribution of DNA among the of *Pf*MCM_N_-WT oligomers, we specifically monitored the absorbance at 490 nm of the fluorescein label of Flc-T40 in AUC sedimentation velocity (Supplementary Figure S3) and equilibrium experiments (Supplementary Figure S4). With *Pf*MCM_N_-WT:Flc-T40, we observe two peaks, *s*_20_ = 1.67 S and 7.77 S (Figure 1C, Supplementary Table S1). The first peak corresponds to unbound Flc-T40, and the second peak is very close to the 7.35 S sedimentation coefficient observed for hexameric apo-*Pf*MCM_N_-WT (Figure 1A, Supplementary Table S1), indicating that the MCM-bound ssDNA specifically associates with hexameric *Pf*MCM_N_-WT. Collectively, the AUC experiments indicate that ssDNA drives hexamerization of *Pf*MCM_N_-WT by preferential association with the hexameric form.

**Figure 2.**
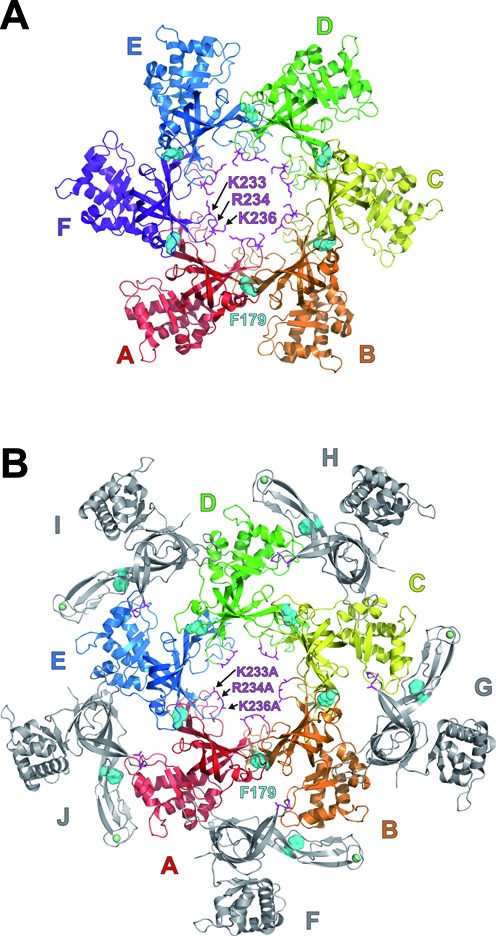
Positions of the mutants of this study in the *Pf*MCM_N_-WT hexamer crystal structure. (**A**) Cartoon representation of the crystal structure of the *Pf*MCM_N_-WT hexameric ring (PDB 4POF (32)) viewed parallel to the ring channel with the Zn-binding subdomains projected into the page. In a full-length protein, the AAA+ ATPase domains (not present) would project out of the page. Each subunit is uniquely colored and labeled. Three positive residues of the β-turn of the purple subunit are shown in magenta stick. The side-chain positions of these residues are likely flexible. The side-chain atoms of a conserved phenylalanine (F179) at the heart of each subunit interface are shown as cyan spheres. In this view from the C-terminal side of the ring, F179 of each subunit interacts with the clockwise subunit. The *Pf*MCM F179 residue aligns with the *MmMcm4(Chaos3)* mutation (37,38) (Supplementary Figure S5). (**B**) Cartoon representation of the X-ray crystal structure of *Pf*MCM_N_-βT. The structure consists of a central pentameric ring with five peripheral subunits. The pentameric ring is grossly similar to the hexameric ring of *Pf*MCM_N_-WT shown in panel A with side-chain atoms of the K233A, R234A, and K236A mutations shown as magenta sticks. The view orients subunit A similar to that shown for subunit A of the hexamer in panel A. Subunits A–E of the central pentameric ring are color-coded and project the Zn-binding domains into the page. The five peripheral subunits are colored grey. Zinc ions are represented as light green spheres. The crystal structure is isomorphic with that of *Pf*MCM_N_-F179A (Supplementary Figure S8A).

We identified three mutants defective for hexamerization (Table 1) to enable further assessment of the role of hexamerization on DNA-binding. The positions of the residues mutated within the wild-type *Pf*MCM_N_ hexamer are shown in Figure 2A. One mutant is a triple-mutant of the *Pf*MCM_N_ β-turn, K233A/R234A/K236A (*Pf*MCM_N_-βT). The second mutant (*Pf*MCM_N_-F179A) involves a residue at the heart of an inter-subunit hydrophobic interface in the *Pf*MCM_N_-WT hexamer crystal structure (32). This phenylalanine aligns in sequence (Supplementary Figure S5) with a phenylalanine of Mcm4 whose mutation, *Mcm4(Chaos3)*, severely disrupts Mcm4:Mcm6 association (39) and has been associated mammary adenocarcinoma (37,38). A third mutation (*Pf*MCM_N_-ΔZFD) removes intersubunit hydrogen bonds normally present as a short antiparallel β-sheet interaction (detailed in the next section). We determined the crystal structure of each mutant (see below and Supplementary Figure S6) to elucidate the molecular details for how the mutations affect hexamerization and also to verify that the mutations do not adversely alter the ssDNA-binding residues identified previously (32).

**Table 1.** Summary of mutants

Name	Solution oligomer	ssDNA-binding	DNA promotes oligomer?
*Pf*MCM_N_-WT (residues 1–256)	Monomer/hexamer	Yes	Yes
*Pf*MCM_N_-βT (1–256; K233A; R234A; K236A)	Monomer/pentamer	No	No
*Pf*MCM_N_-F179A (1–256; F179A)	Monomer	Very weak	No
*Pf*MCM_N_-ΔZFD (1–131; 181–256)	Monomer	No	No

### Structural characterization of hexamerization-defective *Pf*MCM_N_ mutants

**Figure 3.**
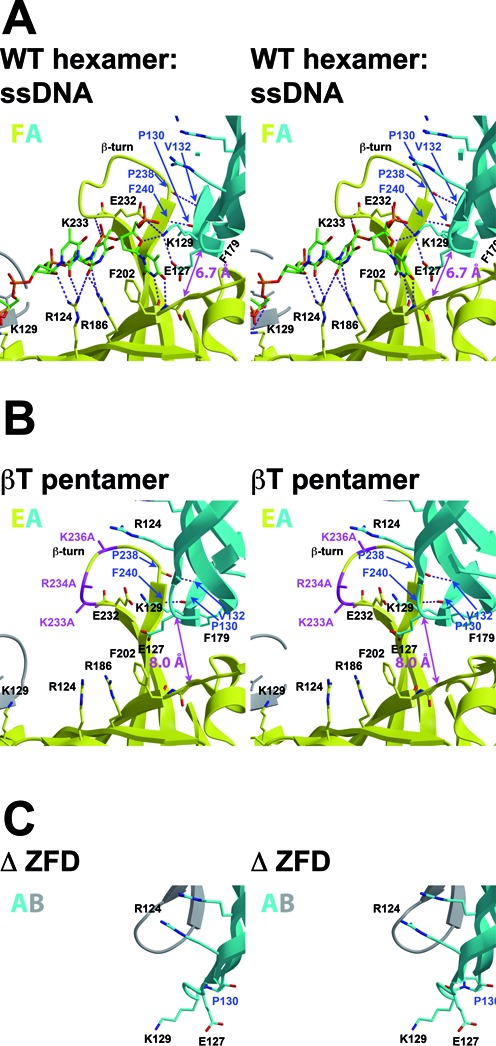
Stereoviews of subunit interfaces in MCM_N_ crystal structures. Binding of ssDNA in the *Pf*MCM_N_-WT hexamer is associated with ‘tight’ subunit interfaces, as demonstrated by the R201 Cα-E127 Cα distance between adjacent subunits shown in magenta (32). The interface with ssDNA-bound (**A**, PDB 4POG (32)) shows a distance <7.0 Å. In contrast, the *Pf*MCM_N_-βT pentameric ring structure (**B**) shows a more open interface with an R201 Cα-E127 Cα distance of 8.0 Å. The hexameric and pentameric ring structures have similar antiparallel intersubunit β-sheet interactions shown in blue (P130 carbonyl–F240 amide; V132 amide–P238 carbonyl). In the Zn-binding subdomain deletion mutant (**C**), a *cis*-proline conformation for P130 and deletion of V132 preclude these interactions.

The crystal structure of *Pf*MCM_N_*-β*T (Supplementary Table S3) consists of a central pentameric ring with five peripheral monomers (Figure 2B, Supplementary Video S1). As in the *Pf*MCM_N_-WT hexamer (Figure 3A), adjacent subunits interact with each other via a short antiparallel β-sheet interaction: P130 carbonyl–F240 amide and V132 amide–P238 carbonyl (Figure 3B). Intriguingly, two of the residues involved in this interaction, P238 and F240, are positioned just following the 3 alanine mutations on the β-turn, providing a straightforward basis for the different oligomeric form of *Pf*MCM_N_-βT than the *Pf*MCM_N_-WT hexamer. In particular, the carbonyl of P238 is notably shifted in *Pf*MCM_N_-βT when compared to *Pf*MCM_N_-WT (Supplementary Figure S7). This shift is not compatible with retaining the specific P238-V132 interaction in a hexamer because the constituent atoms are separated by 4 Å when *Pf*MCM_N_-βT monomers are superimposed on each subunit of the wild-type hexamer (data not shown). The crystal structure of *PfMCM_N_*-F179A (Supplementary Table S3, Supplementary Figure S8A) is isomorphic with that of *Pf*MCM_N_*-β*T and shows a similar central pentamer with adjacent subunits interacting via a short antiparallel β-sheet interaction: P130 carbonyl - F240 amide and V132 amide - P238 carbonyl with a shift in the P238 carbonyl position relative to *Pf*MCM_N_-WT that is similar to *Pf*MCM_N_*-β*T (Supplementary Figure S7). The shifted positions of the P238 carbonyl and the β-turn are likely not directly induced by the F179A mutation because this mutation is not in close proximity (Supplementary Figure S7). Instead, this structure suggests that the wild-type β-turn can be induced to accommodate either pentameric or hexameric ring structures.

We designed the *Pf*MCM_N_-ΔZFD construct to disrupt the short intersubunit antiparallel β-sheet interaction (P130 carbonyl–F240 amide and V132 amide–P238 carbonyl, Figure 3A). Because these interactions exclusively involve main-chain atoms, they cannot be predictably disrupted by side-chain mutagenesis. Instead, we deleted residues 131–181, which deletes the Zn-binding B-subdomain. This deletion removes the V132 amide altogether and requires a different orientation for the P130 carbonyl to accommodate the tight β-hairpin needed to accommodate the deletion. We confirmed that the deletion indeed removed the potential for the short intersubunit β-sheet interaction by determining its crystal structure to 1.55 Å resolution (Supplementary Table S3, Supplementary Figure S8B). The structure shows that P130 adopts a *cis*-proline conformation in order to generate the tight β-hairpin of the deletion (Figure 3C), and thus the carbonyl of this residue is no longer available to interact with a neighboring subunit. Notably, the MSSB residues of *Pf*MCM_N_-ΔZFD are positioned very similar to those of *Pf*MCM_N_-WT when bound to ssDNA (32).

### *Pf*MCM_N_-βT forms a pentamer in solution

**Figure 4.**
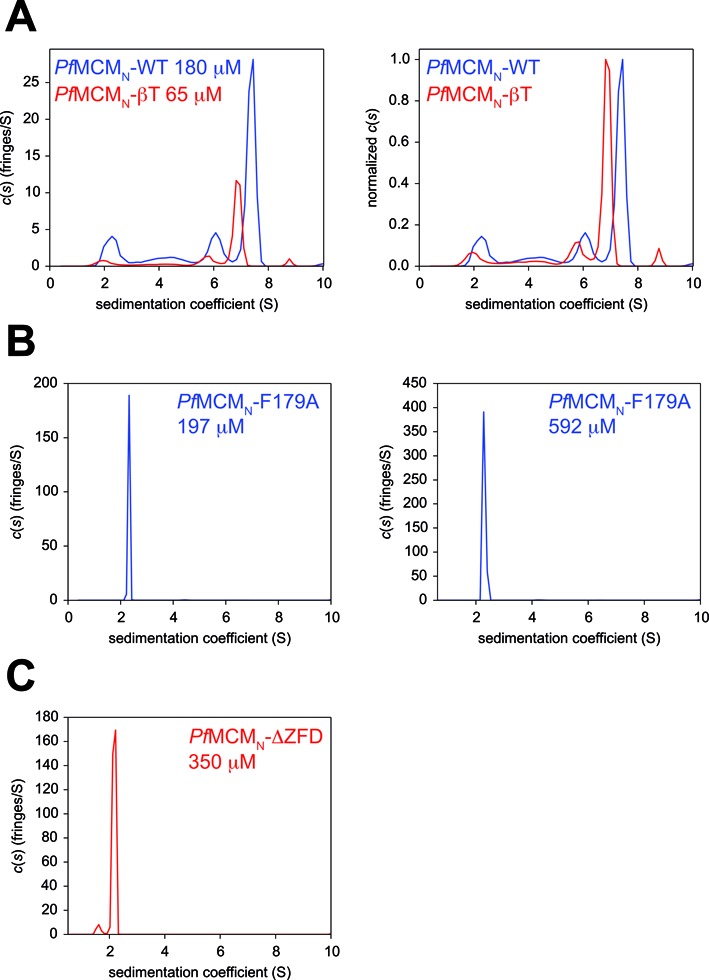
AUC sedimentation coefficient distribution of hexamerization defective mutants. (**A**) AUC sedimentation coefficient distributions of *Pf*MCM_N_-WT and *Pf*MCM_N_-βT show that *Pf*MCM_N_-βT adopts a slower sedimenting oligomer in the 6.5–7.5 S region than that of *Pf*MCM_N_-WT. *Pf*MCM_N_-βT forms this oligomer at a lower protein concentration than *Pf*MCM_N_-WT, and the profiles are normalized (right) for straightforward comparison. (**B**) AUC sedimentation coefficient distribution shows that *Pf*MCM_N_-F179A is a monomer in solution, even at the highest concentration tested (592 μM, right). (**C**) AUC sedimentation coefficient distribution shows that *Pf*MCM_N_-ΔZFD is a monomer in solution.

AUC sedimentation velocity and equilibrium experiments (Supplementary Tables S1 and S2) show that *Pf*MCM_N_-βT most likely adopts a pentameric structure in solution (Figure 4A). This pentameric species is consistent with the central pentamer observed in the *Pf*MCM_N_-βT crystal structure. Analysis with an A_5_ ↔ 5A model (where A_5_ is pentamer and A is monomer) indicates that the protein concentration where 50% is pentameric and 50% is monomeric (*K*_d1–5_ = 8 μM, Supplementary Table S2) is 7-fold lower than that of the *Pf*MCM_N_-WT hexamer (*K*_d1–6_ = 60 μM). AUC sedimentation velocity experiments of *Pf*MCM_N_-F179A (470 μM; Figure 4B, Supplementary Table S1) and *Pf*MCM_N_-ΔZFD (360 μM; Figure 4C, Supplementary Table S1) showed exclusively monomeric species at all concentrations tested, indicating that these mutants do not form detectable oligomers in solution. The pentameric ring structure of *Pf*MCM_N_-F179A observed crystallaographically is not maintained in solution under all conditions tested. This oligomer may form at the high concentrations used for crystallization (which are not feasible for AUC experiments). The *Pf*MCM_N_-F179A pentamer found in the crystal structure is likely stabilized by crystal-packing interactions, including interactions with the peripheral subunits.

### Hexamerization-defective *Pf*MCM_N_ mutants are defective in binding ssDNA

**Figure 5.**
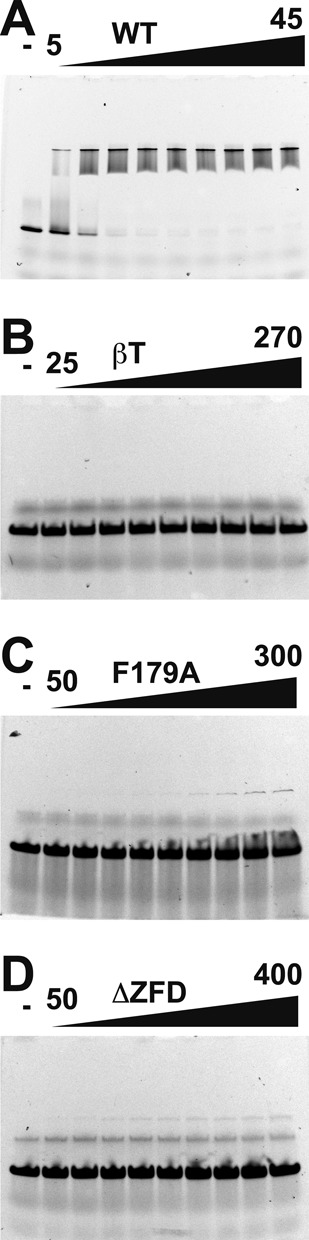
Electrophoretic mobility shift of ssDNA in the presence of *Pf*MCM_N_. Flc-T40 (200 nM) was titrated with the indicated *Pf*MCM_N_ samples: (**A**) *Pf*MCM_N_-WT (0, 5, 10, 15, 20, 25, 30, 35, 40 and 45 μM); (**B**) *Pf*MCM_N_-βT (0, 25, 50, 75, 100, 150, 200, 225, 250 and 270 μM); (**C**) *Pf*MCM_N_-F179A (0, 50, 75, 100, 125, 150, 175, 200, 250 and 300 μM); and (**D**) *Pf*MCM_N_-ΔZFD (0, 50, 75, 100, 150, 200, 250, 300, 350 and 400 μM). The *Pf*MCM_N_-F179A, *Pf*MCM_N_-βT and *Pf*MCM_N_-ΔZFD mutants are all severely defective in binding ssDNA, even in elevated protein concentrations. Lanes marked ‘-’ are loaded with control sample lacking protein.

All of the hexamerization-defective mutants (monomer or pentamer) are completely defective in binding ssDNA in EMSAs (Figure 5A–D). Although these mutants possess an intact MSSB for interaction with DNA, they are nevertheless severely defective in binding ssDNA. The DNA-binding defects observed by EMSA (Figure 5) are consistent with the defects detected by AUC for *Pf*MCM_N_-βT (Supplementary Figure S3B) and for *Pf*MCM_N_-F179 (Supplementary Figure S9). For *Pf*MCM_N_-F179A, a minor shifted band is observed at very high protein concentrations (Figure 5C). AUC sedimentation velocity experiments monitoring the absorbance at 490 nm of the fluorescein label of Flc-T40 show a minor species with *Pf*MCM_N_-F179A that is close to the position of the *Pf*MCM_N_-F179A monomer (Supplementary Figure S9). We therefore consider a ssDNA-bound monomer of *Pf*MCM_N_-F179A to be the most likely species detected by EMSA at high concentration. DNA-binding by this monomeric MCM mutant is therefore very weak, but not entirely ablated. Overall, the severe DNA-binding defects of monomeric and pentameric *Pf*MCM_N_ mutants strongly implicate a role for the hexameric ring structure in binding ssDNA.

The mutations studied here would simultaneously be introduced to all 6 subunits of a symmetric hexameric ring. The phenotypes of 6 simultaneous mutations may be stronger than those of one mutation at a single subunit, such as the single-subunit mutation introduced by *Mcm4(Chaos3)* in eukaryotic Mcm2–7. To qualitatively assess the extent that the *Pf*MCM_N_-F179A mutant can be incorporated into wild-type hexamers that bind DNA, we performed two EMSAs with wild-type and wild-type/mutant mixtures. The concentration of *Pf*MCM_N_-WT was titrated identically for both gels. No mutant was added to the first EMSA, and *Pf*MCM_N_-F179A mutant was added to maintain a constant total protein in the second EMSA. If *Pf*MCM_N_-F179A has no capacity to interact with wild-type, then the two gels should look equivalent. If *Pf*MCM_N_-F179A appreciably interacts with wild-type, the binding trace could differ (either increase or decrease). These titrations look very similar (Supplementary Figure S10), suggesting that the level of DNA-binding can be predicted solely by the concentration of wild-type protein. Although not conclusive, the simplest rationalization of these results is that *Pf*MCM_N_-F179A does not significantly incorporate into mixed hexamers with *Pf*MCM_N_-WT.

## DISCUSSION

Although all subunits of the *PfMCM_N_* hexameric ring are chemically identical, ssDNA is not present equivalently at each subunit of the *PfMCM_N_*:ssDNA crystal structure (32). Instead, the structure shows that ssDNA specifically binds at subunits with smaller intersubunit distances. Thus, ssDNA-binding is determined not just by residue identities, but also by the relative orientations of these residues on adjacent subunits. Here, we extend these findings to show that ssDNA-binding is indeed exquisitely sensitive to establishing a specific intersubunit interface within a hexameric ring. *Pf*MCM_N_-WT cooperatively assembles on ssDNA to yield a hexamer, and mutants defective for hexamerization do not bind ssDNA. Such binding requires the specific geometry of a hexameric ring– a mutant that forms a pentameric ring does not bind DNA despite retaining the residues needed to directly interact with DNA.

The hexamerization of *PfMCM_N_*-WT is concentration-dependent, consistent with previous observation that the protein elutes as a monomer during size-exclusion chromatography but crystallizes as a hexamer (32). The presence of ssDNA promotes hexamer stability, likely by direct interactions with multiple subunits, as observed in the crystal structure (32). Likewise, T7gp4 hexamerization is stimulated by the presence of ssDNA (59,60). The oligomeric structures of other hexameric helicases are also stabilized by DNA or nucleotide co-factors. For instance, RepA requires Mg^2+^, nucleotide, or oligonucleotide to stabilize the hexameric ring (61–64).

Mutants that are defective for hexamerization are severely defective in binding ssDNA. All of these mutants (*Pf*MCM_N_-ΔZFD, *Pf*MCM_N_-F179A and *Pf*MCM_N_-βT) have wild-type MSSB motifs available for interaction with ssDNA, but no binding is observed. The *Pf*MCM_N_-ΔZFD and *Pf*MCM_N_-F179A mutants are exclusively monomeric at all concentrations tested. The monomeric preference of *Pf*MCM_N_-F179A is consistent with the ablation of Mcm4:Mcm6 association in *Mm*Mcm4 F345I (39) and also the monomer observed by size-exclusion chromatography for the similarly positioned L189D/D191R double-mutant of *Sso*MCM (26). The *Pf*MCM_N_-βT mutant forms a pentameric ring that does not bind ssDNA. While the intersubunit configuration appears quite malleable for the *PfMCM_N_*-WT hexamer, a pentameric ring has fewer degrees of freedom. A pentamer, therefore, may not be able to attain the precise interface needed to bind ssDNA. The requirement for a hexameric ring of MCM_N_ to bind DNA is similar to that of the bacteriophage T7gp4 hexameric helicase. T7gp4 is observed in hexameric (65) or heptameric (66) forms, but only the hexamer can bind DNA (59).

Our results with *Pf*MCM_N_-βT demonstrate an important role for the MCM β-turn in oligomerization. This module may have additional roles in stabilizing association with DNA. The crystal structure of *Pf*MCM_N_:ssDNA (32) showed no direct interaction between the β-turn of *Pf*MCM_N_ and ssDNA, and the β-turn single mutant *Pf*MCM_N_-K233A was not significantly impaired in binding ssDNA (32). However, a less direct role for the β-turn positive residues in stabilizing association between MCM and ssDNA could occur by overall charge neutralization. The positive residues of the β-turn are very likely to interact directly with dsDNA if the hexameric ring encircles dsDNA because the N-terminal domain β-turn is the structural module that projects farthest into the ring channel (23) (Figure 2A). A role for positive residues on the β-turn in binding dsDNA is well-supported biochemically (9,23).

A requirement for specifically oriented MCM amino-terminal domains for productive DNA binding is conceptually analogous to the requirement for precisely positioned ATPase domains for ATP hydrolysis. The MCM AAA+ ATPase active site is composed of two adjacent subunits, with Walker A and Walker B motif residues of one subunit and residues such as the arginine finger on the adjacent subunit (12,67,68). Together the two subunits adopt a very precise configuration to hydrolyze ATP (2). Similarly, MSSB motifs of adjacent subunits need to attain a precise configuration to enable binding to ssDNA. This relationship implies a straightforward method for the MCM AAA+ ATPase domains to modulate ssDNA-binding at the N-terminal domain. Progression of the AAA+ domains through distinct configurations via the ATPase cycle could directly alter the intersubunit configuration at the associated N-terminal domains and thus influence ssDNA-binding affinity. Further, the AAA+ domains may partially compensate for oligomerization defects at the N-terminal domain. The association of accessory factors may also place the MCM ring in specific conformations that favor (or disfavor) binding to ssDNA.

Although we observe that the *Pf*MCM_N_-F179A mutant does not hexamerize under any concentration tested, the homologous residue mutation introduced by *Mcm4(Chaos3), Mm*Mcm4 F345I, likely permits formation of a hexamer of Mcm2–7 that is not completely defective because the allele is viable (38). The *Mm*Mcm4 F345 residue is very likely at the heart of an intersubunit interface with *Mm*Mcm6, consistent with disruption of Mcm4:Mcm6 association for the *Mm*Mcm4 F345I mutant (39) and also the observed position of the corresponding *Saccharomyces cerevisiae* (*Sc*) Mcm4 residue, F391, at an interface with *Sc*Mcm6 in the structure of *Sc*Mcm2–7 (69). Among the many possible consequences of perturbing the Mcm4:Mcm6 interface by *Mm*Mcm4 F345I, the correlation of intersubunit configuration with ssDNA-binding that we describe here raises the possibility that binding to ssDNA could be affected.

## Supplementary Material

SUPPLEMENTARY DATA

## ACCESSION NUMBERS

PDB: 4YWL, 4YWM and 4YWK.
