# Supplementary material for: MCM ring hexamerization is a prerequisite for DNA-binding
Source: Nucleic Acids Res. 2015 Sep 13;43(19):9553–63. doi: 10.1093/nar/gkv914 (PMC4627082; doi:10.1093/nar/gkv914)
Supplement: SUPPLEMENTARY DATA [file supp_43_19_9553__index.html]

MCM ring hexamerization is a prerequisite for DNA-binding — MCM ring hexamerization is a prerequisite for DNA-binding — SUPPLEMENTARY DATA 

# MCM ring hexamerization is a prerequisite for DNA-binding

## SUPPLEMENTARY DATA

- SUPPLEMENTARY DATA
- SUPPLEMENTARY DATA
- SUPPLEMENTARY DATA
