# Supplementary material for: MCM ring hexamerization is a prerequisite for DNA-binding
Source: Nucleic Acids Res. 2015 Sep 13;43(19):9553–63. doi: 10.1093/nar/gkv914 (PMC4627082; doi:10.1093/nar/gkv914)
Supplement: SUPPLEMENTARY DATA [file supp_gkv914_nar-01263-m-2015-File007.pdf]

MCM ring hexamerization is a prerequisite for DNA-binding

Clifford A. Froelich[1], Amanda Nourse[2], and Eric J. Enemark[1*]

[1]Department of Structural Biology, St Jude Children's Research Hospital, 262 Danny Thomas Place, Mail Stop 311, Memphis, TN 38105, USA

[2]Molecular Interaction Analysis Shared Resource, St. Jude Children's Research Hospital, 262 Danny Thomas Place, Mail Stop 311, Memphis, TN 38105, USA

* To whom correspondence should be addressed.

Phone: +1 901-595-6178; FAX: +1 901-595-3032; Email: eric.enemark@stjude.org

Supplementary Materials

Supplementary Figure S1. Isotherm of the signal-average s-values, s_w, of the total sedimenting system derived from integration of the complete c(s) distributions of *Pf*MCM$_N$-WT at various concentrations

Supplementary Figure S2. AUC sedimentation coefficient distribution of *Pf*MCM$_N$-WT:ssDNA

Supplementary Figure S3. AUC Sedimentation Velocity for FLC-ssDNA:protein mixtures

Supplementary Figure S4. AUC Sedimentation Equilibrium

Supplementary Figure S5. Sequence Alignment of *Pf*MCM$_N$-F179 and Mcm4(Chaos3)

Supplementary Figure S6. Subdomain architecture of *Pf*MCM$_N$ and mutation positions

Supplementary Figure S7. The structures of a constituent subunit of pentameric MCM and hexameric MCM are very similar except for the β-turn

Supplementary Figure S8. X-ray Crystal Structures of *Pf*MCM$_N$-F179A and *Pf*MCM$_N$-ΔZFD

Supplementary Figure S9. AUC sedimentation coefficient distribution analysis of *Pf*MCM_N-F179A in the presence of ssDNA

Supplementary Figure S10. Comparison of DNA-binding by *Pf*MCM_N-WT and *Pf*MCM_N-WT/*Pf*MCM_N-F179A mixtures

Supplementary Table S1. Analytical Ultracentrifugation Sedimentation Velocity

Supplementary Table S2. Analytical Ultracentrifugation Sedimentation Equilibrium

Supplementary Table S3. Crystallographic Statistics

Supplementary References

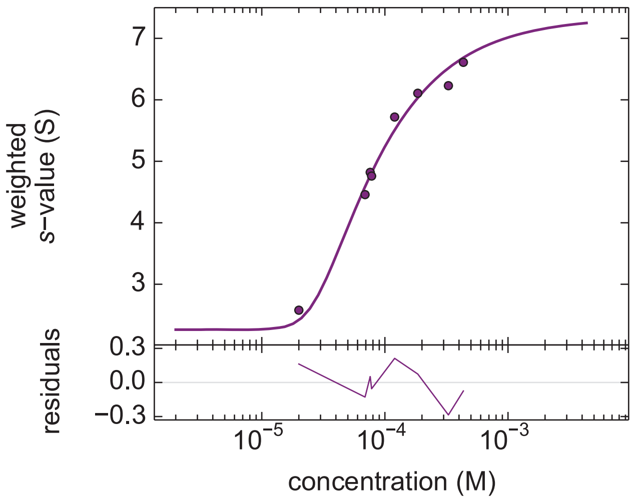

Supplementary Figure S1. Isotherm of the signal-average s-values, s_w, of the total sedimenting system derived from integration of the complete c(s) distributions of *Pf*MCM_N-WT at various concentrations. The solid line is the fitted isotherm of a reversible monomer-hexamer self-association system. $K_{D1\text{-}6}$ is 58.0 μM. The s_w-values of pure monomer (2.4 S) and pure hexamer (7.8 S) were assumed and not experimentally determined.

A

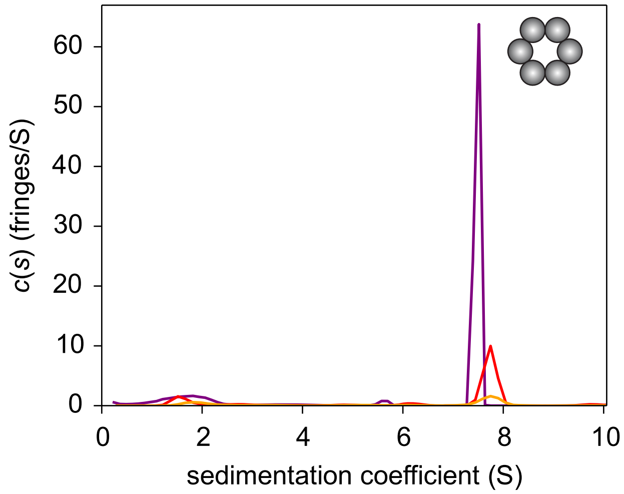

B

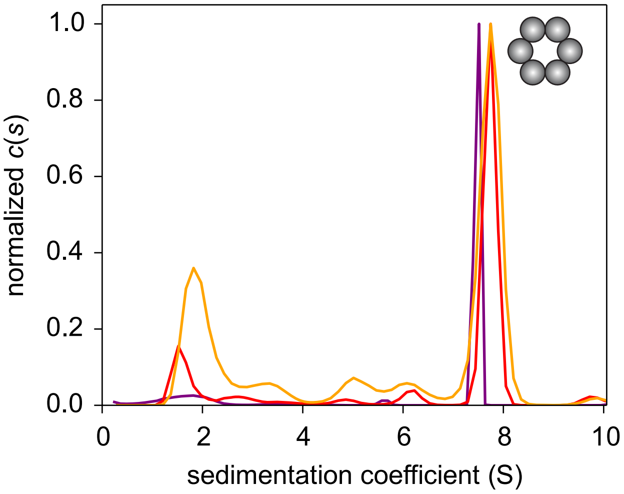

108 µM *Pf*MCM_N-WT + 20 µM DNA (Total 128 µM)

38 µM *Pf*MCM_N-WT + 7 µM ssDNA (Total 45 µM)

12.7 µM *Pf*MCM_N-WT + 2.3 µM ssDNA (Total 15 µM)

Supplementary Figure S2. AUC sedimentation coefficient distribution of *Pf*MCM_N-WT:ssDNA. **A**) Higher concentrations show a larger fraction of hexameric *Pf*MCM_N-WT. **B**) Each distribution is normalized to facilitate comparison. The fraction of low molecular weight species increases with decreasing concentration.

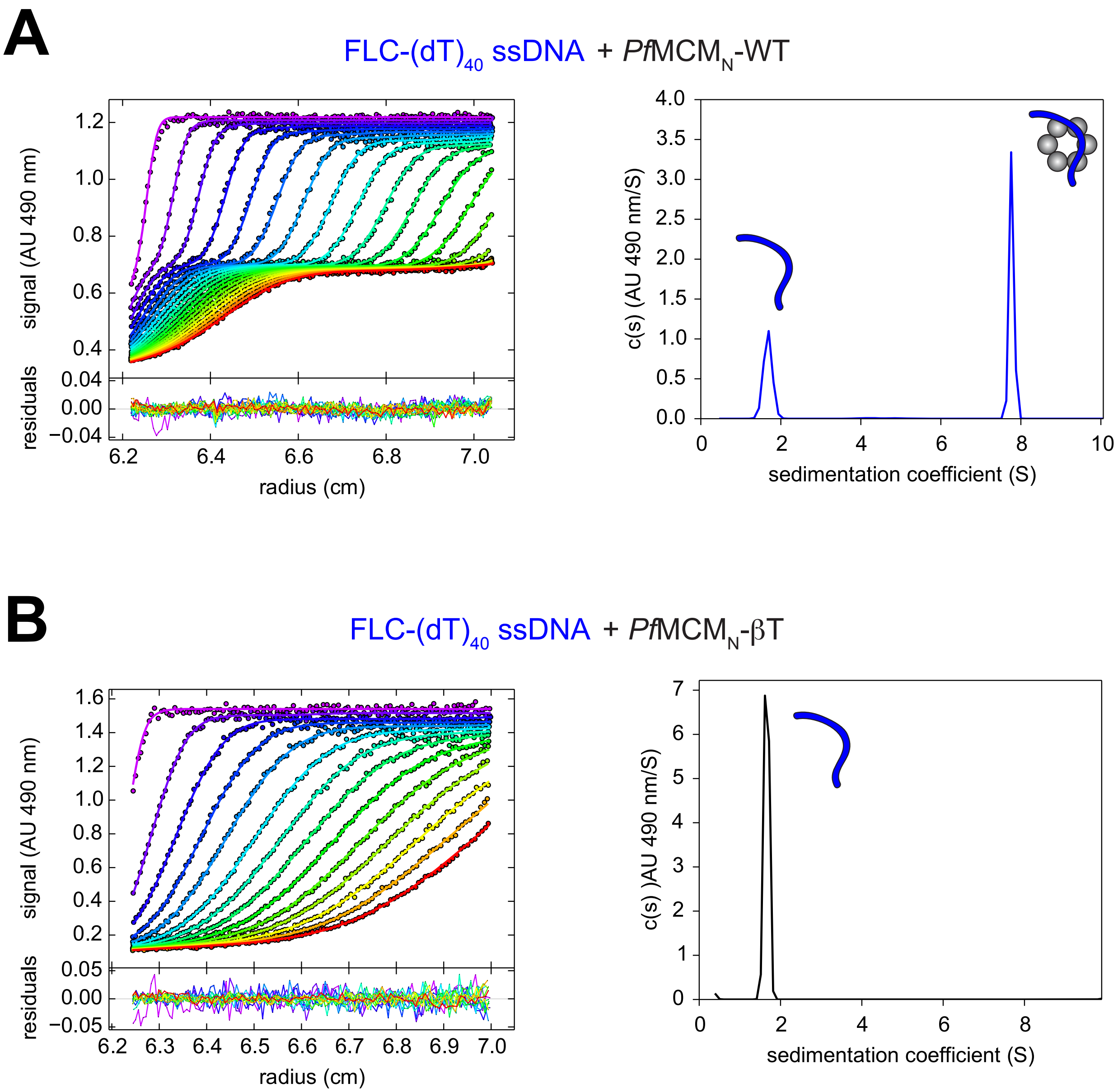

Supplementary Figure S3. AUC Sedimentation Velocity for FLC-ssDNA:protein mixtures. **A**) FLC-DNA mixed with *Pf*MCM$_N$-WT. The radial absorbance profiles at 490 nm as a function of time indicated by color temperature is shown at left and the best-fit sedimentation velocity profiles fitted to the continuous sedimentation coefficient distribution model c(s) are shown at right. Residuals of the fits are shown in the lower left panel. The total concentration of the *Pf*MCM$_N$-WT:Flc-T40 mixture was (54.6 μM:10 μM). **B**) FLC-DNA mixed with *Pf*MCM$_N$-βT. The radial absorbance profile at 490 nm as a function of time indicated by color temperature is shown at left and the best-fit sedimentation velocity profiles fitted to the continuous sedimentation coefficient distribution model c(s) are shown at right. Residuals of the fits are shown in the lower left panel. The total concentration of the *Pf*MCM$_N$-βT:Flc-T40 mixture was (92.8 μM:17 μM). The experiments were conducted in 20 mM HEPES pH 7.6, 200 mM NaCl and 5 mM BME buffer at 20 ºC and at a rotor speed of 50,000 rpm. The s-values of the species are listed in Supplementary Table S1.

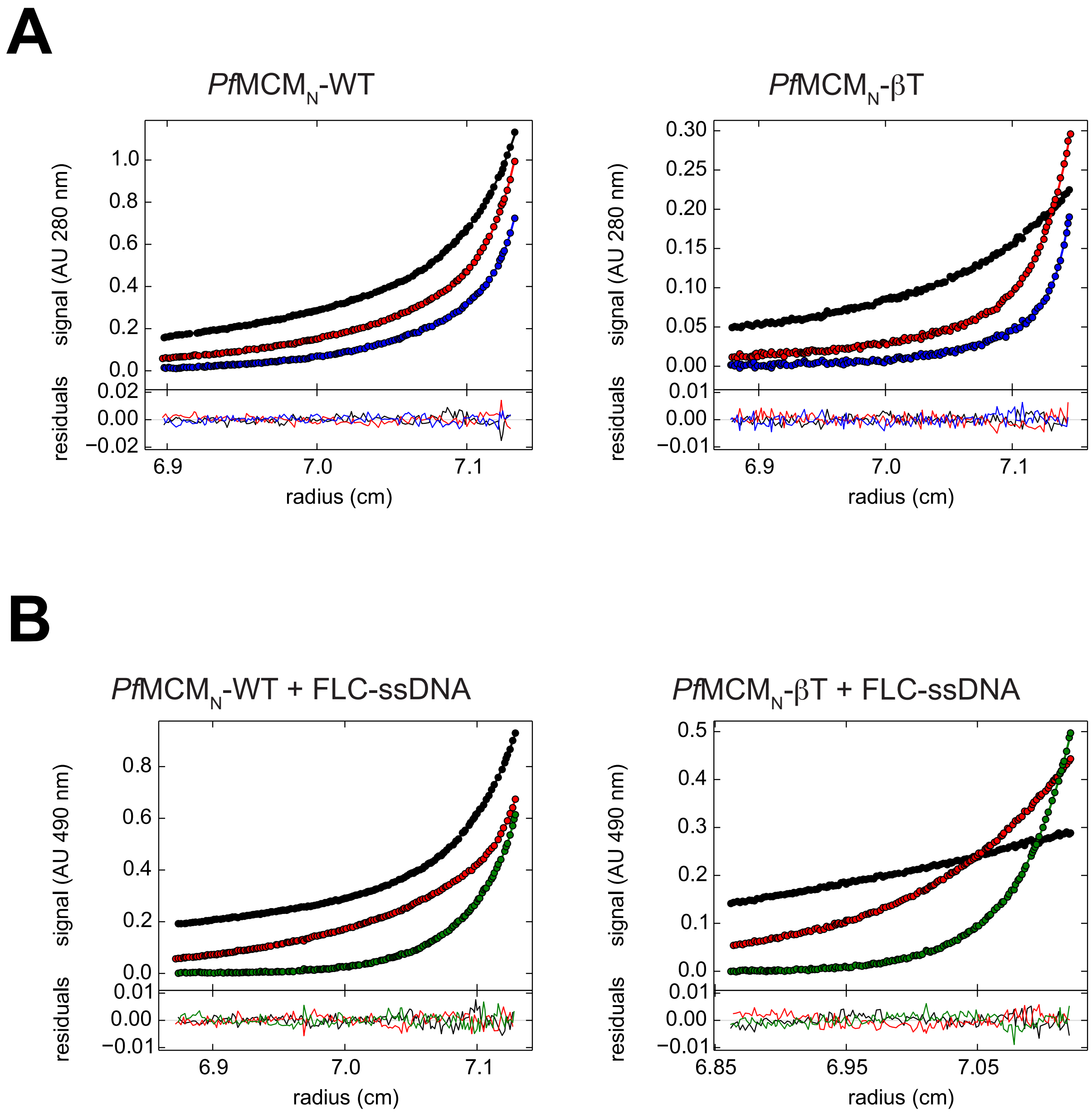

Supplementary Figure S4. AUC Sedimentation Equilibrium. **A**) Absorbance scans at 280 nm at equilibrium are plotted versus the distance from the axis of rotation (radius). The proteins were centrifuged in 20 mM HEPES pH 7.6, 200 mM NaCl and 5 mM βME buffer at 20 °C at increasing speeds of 15,000 rpm (black), 20,000 rpm (red), and 25,000 rpm (blue) for *Pf*MCM$_N$-WT and 10,000 rpm (black), 18,000 rpm (red) and 25,000 rpm (blue) for *Pf*MCM$_N$-βT. Shown are *Pf*MCM$_N$-WT at 35 μM and *Pf*MCM$_N$-βT at 12 μM. The solid lines represent the global nonlinear least squares best-fit of all the data sets to a reversible monomer-hexamer (1↔6) self-association model for *Pf*MCM$_N$-WT or a monomer-pentamer (1↔5) self-association model for *Pf*MCM$_N$-βT. **B**) Diluted mixtures of protein:Flc-T40 were centrifuged in the same buffer at 4 °C at increasing speeds of 12,000 rpm (black), 21,000 rpm (red), and 35,000 rpm (green). Shown are *Pf*MCM$_N$-WT:Flc-T40 (41.5 μM:7.6 μM) and *Pf*MCM$_N$-βT:Flc-T40 (19.1 μM:3.5 μM). The solid lines represent the global nonlinear least squares best-fit of all the data sets to a reversible single site hetero-association model (A+B↔AB) with A the monomer Flc-T40 species and B the hexamer species for *Pf*MCM$_N$-WT. Data of *Pf*MCM$_N$-βT were fitted to the single species model. The results are listed in Table S2.

*Pf*MCM	D	V	N	K	S	S	F	V	N	F	Q	S	F	R	I	Q	D	R	P	E	T	L	194
*Sso*MCM	I	P	E	K	T	K	L	I	D	W	Q	K	A	V	I	Q	E	R	P	E	E	V	204
*Mt*MCM	L	Q	D	E	S	E	F	L	D	T	Q	T	L	K	L	Q	E	P	L	E	N	L	186
*Ap*MCM	V	R	D	K	S	L	Y	I	D	W	Q	K	I	M	V	Q	E	R	P	E	D	V	208
*Sc*Mcm2	N	G	E	K	T	V	Y	R	N	Y	Q	R	V	T	L	Q	E	A	P	G	T	V	397
*Dm*Mcm2	N	M	E	Q	T	L	Y	R	N	Y	Q	K	I	T	L	Q	E	S	P	G	R	I	370
*Xl*Mcm2	N	M	E	E	T	V	Y	Q	N	Y	Q	R	I	T	I	Q	E	S	P	G	K	V	370
*Hs*Mcm2	N	M	E	E	T	I	Y	Q	N	Y	Q	R	I	R	I	Q	E	S	P	G	K	V	385
*Mm*Mcm2	N	M	E	E	T	I	Y	Q	N	Y	Q	R	I	R	I	Q	E	S	P	G	K	V	385
*At*Mcm2	N	V	E	Q	T	I	Y	R	N	Y	Q	K	L	T	I	Q	E	S	P	G	T	V	411
*Sp*Mcm2	N	S	E	R	T	V	Y	N	N	Y	Q	R	I	T	L	Q	E	S	P	G	T	V	390
*Dr*Mcm2	N	M	E	Q	T	V	Y	Q	N	Y	Q	R	I	T	I	Q	E	S	P	G	K	V	373
*Gi*Mcm42	S	T	S	G	T	T	Y	E	D	F	Q	R	I	T	V	Q	E	P	P	N	S	V	450
*Ec*Mcm2	N	T	S	E	T	V	Y	K	D	F	Q	K	L	T	I	Q	E	I	P	G	S	V	290
*Sc*Mcm3	E	Y	G	Y	S	T	F	I	D	H	Q	R	I	T	V	Q	E	M	P	E	M	A	265
*Dm*Mcm3	E	Y	G	L	S	V	Y	K	D	D	Q	T	L	T	I	Q	E	M	P	E	K	A	205
*Xl*Mcm3	E	Y	G	L	S	T	Y	K	D	H	Q	T	L	S	I	Q	E	M	P	E	K	A	208
*Hs*Mcm3	E	Y	G	L	S	V	Y	K	D	H	Q	T	I	T	I	Q	E	M	P	E	K	A	208
*Mm*Mcm3	E	Y	G	L	S	V	Y	K	D	H	Q	T	I	T	I	Q	E	M	P	E	K	A	208
*At*Mcm3	E	Y	G	L	C	K	Y	K	D	H	Q	T	L	S	I	Q	E	V	P	E	N	A	199
*Sp*Mcm3	E	F	G	F	S	T	F	R	D	H	Q	S	I	S	L	Q	E	M	P	E	R	A	213
*Dr*Mcm3	E	F	G	L	S	V	Y	K	D	H	Q	T	I	T	I	Q	E	M	P	E	K	A	207
*Gi*Mcm43	E	Y	G	L	S	L	F	D	S	V	Q	K	I	L	V	Q	D	H	P	E	Y	V	237
*Ec*Mcm3	E	F	G	L	S	E	Y	F	D	Y	Q	T	V	V	L	Q	E	M	P	E	K	A	191
*Sc*Mcm4	I	H	N	R	C	S	F	A	D	K	Q	V	I	K	L	Q	E	T	P	D	F	V	406
*Dm*Mcm4	I	H	N	R	S	E	F	T	D	K	Q	L	V	K	L	Q	E	S	P	D	D	M	364
*Xl*Mcm4	I	H	N	R	S	M	F	S	D	K	Q	M	I	K	L	Q	E	S	P	E	D	M	361
*Hs*Mcm4	I	H	N	R	S	L	F	S	D	K	Q	M	I	K	L	Q	E	S	P	E	D	M	361
*Mm*Mcm4	I	H	N	R	S	F	F	S	D	K	Q	M	I	K	L	Q	E	S	P	E	D	M	360
*At*Mcm4	V	H	N	R	C	R	F	A	D	K	Q	I	V	R	L	Q	E	T	P	D	E	I	329
*Sp*Mcm4	I	H	N	R	S	E	F	A	D	K	Q	V	I	K	L	Q	E	T	P	D	V	V	383
*Dr*Mcm4	V	H	N	R	S	V	F	S	D	K	Q	M	I	K	L	Q	E	S	P	E	D	M	343
*Gi*Mcm44	V	H	N	M	C	T	F	I	D	K	R	V	I	K	I	Q	E	A	P	D	Q	L	275
*Ec*Mcm4	V	H	N	A	S	E	F	E	D	K	Q	V	V	R	I	Q	E	L	P	E	G	I	232
*Sc*Mcm5	I	H	E	S	S	K	F	I	D	Q	Q	F	L	K	L	Q	E	I	P	E	L	V	265
*Dm*Mcm5	M	P	D	K	C	K	C	V	D	F	Q	T	L	K	L	Q	E	L	P	D	F	V	231
*Xl*Mcm5	I	P	D	K	C	K	C	V	D	F	Q	T	L	K	L	Q	E	S	P	D	A	V	237
*Hs*Mcm5	M	P	D	K	C	K	C	V	D	F	Q	T	L	K	L	Q	E	L	P	D	A	V	236
*Mm*Mcm5	M	P	D	K	C	K	C	V	D	F	Q	T	L	K	L	Q	E	L	P	D	A	V	236
*At*Mcm5	V	P	D	R	S	Q	Y	V	D	Q	Q	T	L	K	L	Q	E	N	P	E	D	V	231
*Sp*Mcm5	D	H	S	K	S	T	F	I	D	Q	Q	V	L	K	L	Q	E	A	P	D	M	V	232
*Dr*Mcm5	I	P	D	R	C	V	C	V	D	F	Q	T	Q	R	L	Q	E	A	P	D	A	V	237
*Gi*Mcm45	N	P	H	S	C	V	Y	I	D	Q	Q	S	I	K	L	Q	D	I	P	G	D	M	224
*Ec*Mcm5	I	P	E	K	S	Q	V	I	D	V	Q	Y	V	K	I	Q	E	F	F	E	D	I	202
*Sc*Mcm6	N	V	T	R	S	R	F	L	D	W	Q	K	V	R	I	Q	E	N	A	N	E	I	368
*Dm*Mcm6	D	V	E	K	S	L	F	L	D	F	Q	K	I	R	I	Q	E	T	Q	A	E	L	209
*Xl*Mcm6_	D	T	N	K	S	R	F	V	D	F	Q	K	V	R	I	Q	E	T	Q	A	E	L	217
*Hs*Mcm6	D	T	N	K	S	R	F	V	D	F	Q	K	V	R	I	Q	E	T	Q	A	E	L	215
*Mm*Mcm6	D	T	N	K	S	R	F	V	D	F	Q	K	V	R	I	Q	E	T	Q	A	E	L	215
*At*Mcm6	L	R	Q	E	S	K	F	A	D	W	Q	R	V	R	M	Q	E	T	S	K	E	I	212
*Sp*Mcm6	N	I	S	Q	S	S	F	Q	D	W	Q	K	V	R	I	Q	E	N	S	N	E	I	290
*Dr*Mcm6	D	T	N	K	S	K	F	I	D	F	Q	K	L	R	I	Q	E	T	Q	A	E	L	213
*Gi*Mcm46	L	T	D	Q	C	T	F	A	D	T	Q	R	V	R	L	Q	E	S	I	A	D	T	234
*Ec*Mcm6	D	I	D	K	S	K	F	L	N	W	Q	R	I	H	V	Q	E	N	T	E	E	I	204
*Sc*Mcm7	S	T	R	A	S	K	F	S	A	F	Q	E	C	K	I	Q	E	L	S	Q	Q	V	322
*Dm*Mcm7	Q	T	R	G	S	K	F	V	K	F	Q	E	V	K	M	Q	E	H	S	D	Q	V	244
*Xl*Mcm7	Q	T	R	G	S	K	F	I	K	F	Q	E	L	K	I	Q	E	H	S	D	Q	V	243
*Hs*Mcm7	Q	T	R	G	S	R	F	I	K	F	Q	E	M	K	M	Q	E	H	S	D	Q	V	244
*Mm*Mcm7	Q	T	R	G	S	K	F	V	K	F	Q	E	M	K	I	Q	E	H	S	D	Q	V	244
*At*Mcm7	Q	L	R	A	S	K	F	L	K	F	Q	E	A	K	M	Q	E	L	A	E	H	V	238
*Sp*Mcm7	S	T	R	A	S	K	F	L	P	F	Q	E	V	K	I	Q	E	L	T	N	Q	V	265
*Dr*Mcm7	Q	T	R	G	S	K	F	I	K	F	Q	E	L	R	I	Q	E	H	S	D	Q	V	244
*Gi*Mcm47	N	T	K	R	S	V	I	N	S	L	Y	I	A	I	I	Q	E	L	P	V	E	I	213
*Ec*Mcm7	V	T	R	G	S	K	F	I	K	H	Q	T	V	Y	M	Q	E	L	T	G	D	I	242

Supplementary Figure S5. Sequence Alignement of *Pf*MCM$_N$-F179 and Mcm4(Chaos3). The aligned position of *Pf*MCM$_N$-F179 and Mcm4(Chaos3) are shaded magenta. Residues that are conserved by Mcm family are shaded cyan, and residues that are strongly conserved among all Mcm subunits are shaded dark blue. The position of the Mcm4(Chaos3) mutation, *Mm*Mcm4 F345I (1-3), is circled.

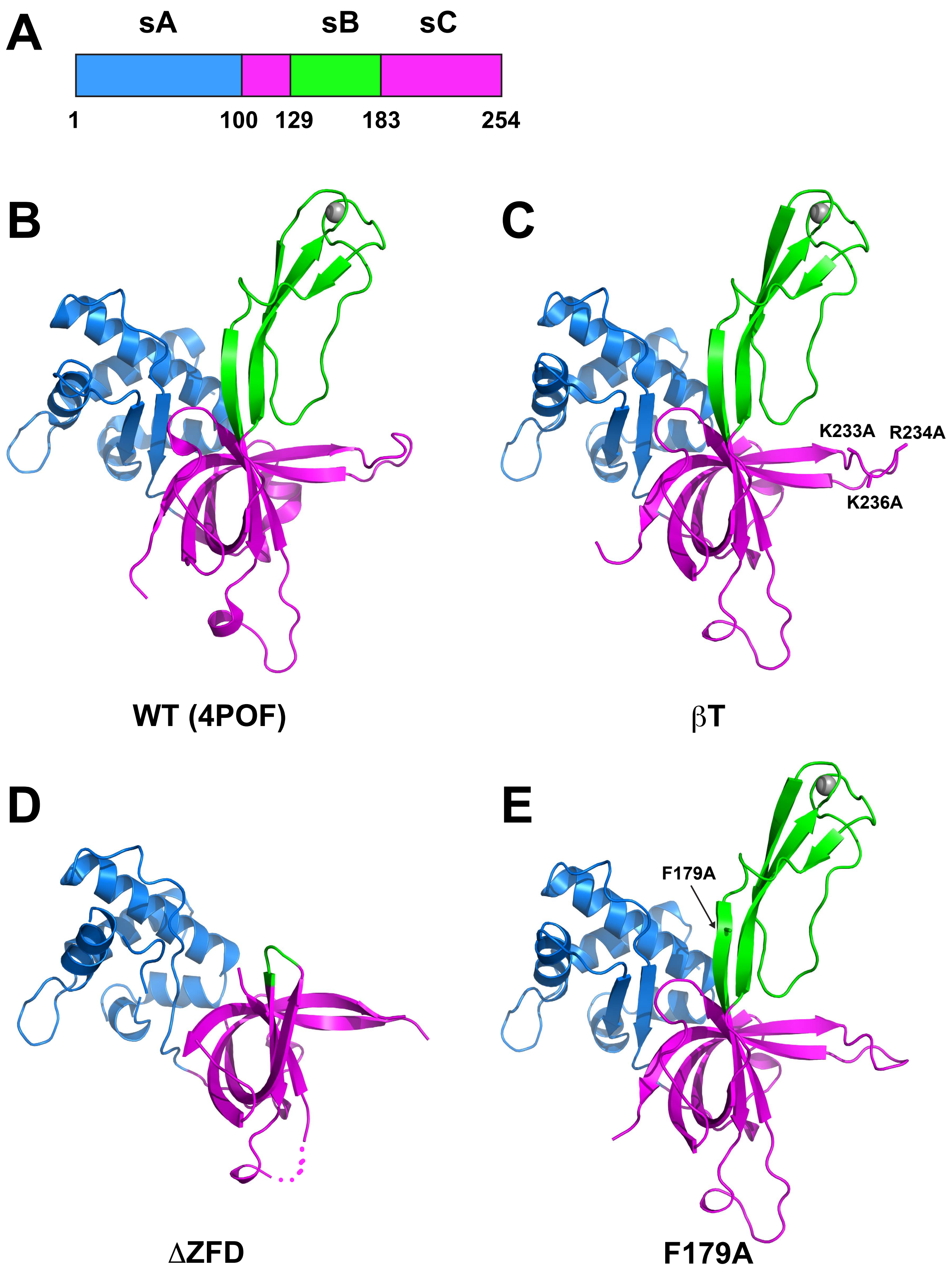

Supplementary Figure S6. Subdomain architecture of *Pf*MCM$_N$ and mutation positions. **A**) The MCM N-terminal domain has three structural subdomains (4): a helical bundle subdomain-A (blue), a zinc-binding subdomain-B (green), and an OB-fold subdomain-C (magenta). Residue numbers for the *Pf*MCM$_N$ subdomain boundaries are provided. **B**) The structure of one *Pf*MCM$_N$-WT subunit (5) with subdomains colored as in panel A. **C**) The structure of one *Pf*MCM$_N$-βT subunit with subdomains colored as in panel A and 3 mutation positions labeled. **D**) The structure of one *Pf*MCM$_N$-ΔZFD subunit with subdomains colored as in panel A. **E**) The structure of one *Pf*MCM$_N$-F179A subunit with subdomains colored as in panel A and mutation position labeled.

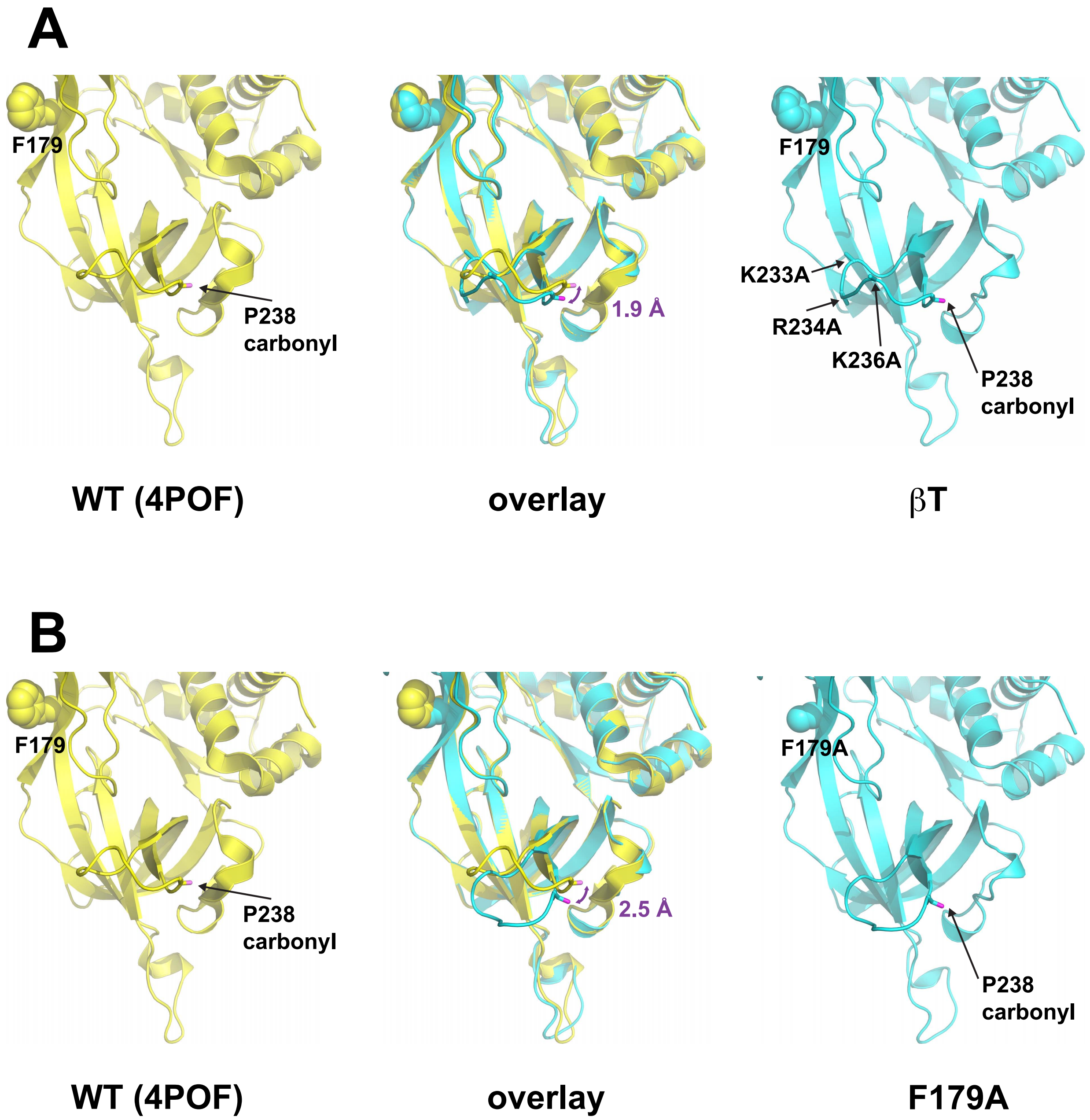

Supplementary Figure S7. The structures of a constituent subunit of pentameric MCM and hexameric MCM are very similar except for the β-turn. **A**) chain A of the wild-type hexamer is compared to chain A of the *Pf*MCM$_N$-βT pentamer. The β-turn is notably shifting, including the carbonyl group of P238, which is involved in intersubunit interactions (Figure 3). **B**) chain A of the wild-type hexamer is compared to chain A of the *Pf*MCM$_N$-F179A pentamer. The β-turn is notably shifting, including the carbonyl group of P238, which is involved in intersubunit interactions (Figure 3). This shift does not likely result directly from the F179A mutation (shown in spheres), which is distant from the β-turn.

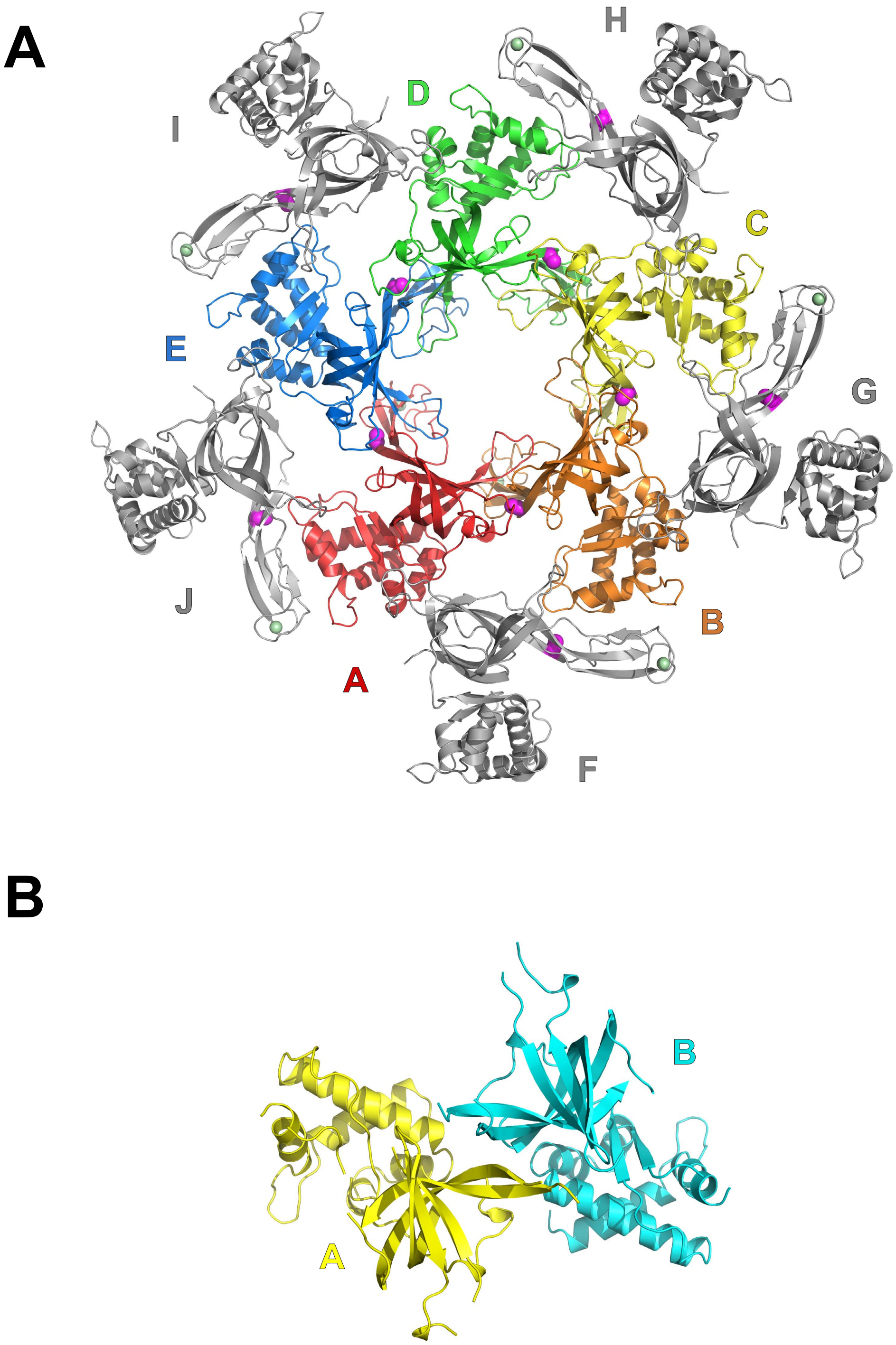

Supplementary Figure S8. X-ray Crystal Structures of *Pf*MCM$_N$-F179A and *Pf*MCM$_N$-ΔZFD. **A**) X-ray Crystal Structure of *Pf*MCM$_N$-F179A. The structure shows a central pentameric ring with five peripheral subunits. Subunits A-E of the central pentameric ring are color-coded and project the Zn-binding domains into the page. The five peripheral subunits are colored grey. The F179A residue of each subunit is colored magenta with the alanine side-chain atom shown in sphere representation. Zinc ions are represented as light green spheres. The crystal structure is isomorphic with that of *Pf*MCM$_N$-βT and is shown in a view equivalent to that of Figure 2. **B**) X-ray Crystal Structure of *Pf*MCM$_N$-ΔZFD. The structure consists of two crystallographically unique subunits colored yellow and cyan. The two subunits are related by a non-crystallographic 2-fold axis. The view is approximately parallel to the non-crystallographic 2-fold axis.

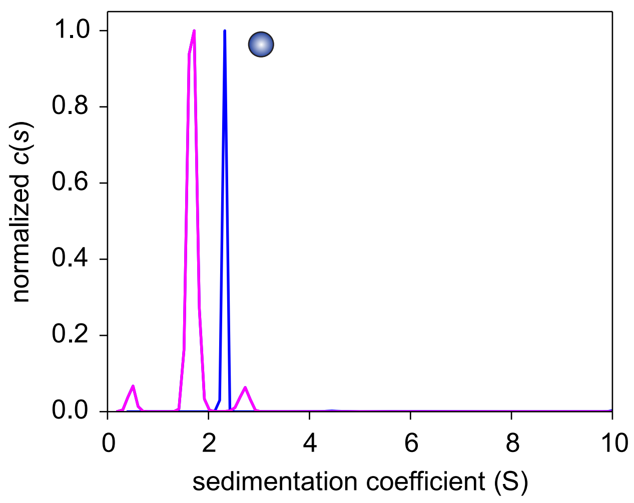

125 µM *Pf*MCM$_N$-F179A + 23 µM FLC-T40
(Total 148 µM; OD 490 nm)

197 µM *Pf*MCM$_N$-F179A (Fringes)

Supplementary Figure S9. AUC sedimentation coefficient distribution analysis of *Pf*MCM$_N$-F179A in the presence of ssDNA. When monitoring fringes (blue), a single peak is observed consistent with a monomer. When specifically monitoring the fluorescein label of FLC-T40 at 490 nm (magenta), the major peak is consistent with the position observed for free (unbound) ssDNA. A minor peak is observed at a slightly larger mass than the monomeric peak of of *Pf*MCM$_N$-F179A by itself. The mass change is consistent with the added mass of ssDNA. Our best assessment is therefore that the minor species detected consists of a monomer of of *Pf*MCM$_N$-F179A bound to FLC-T40.

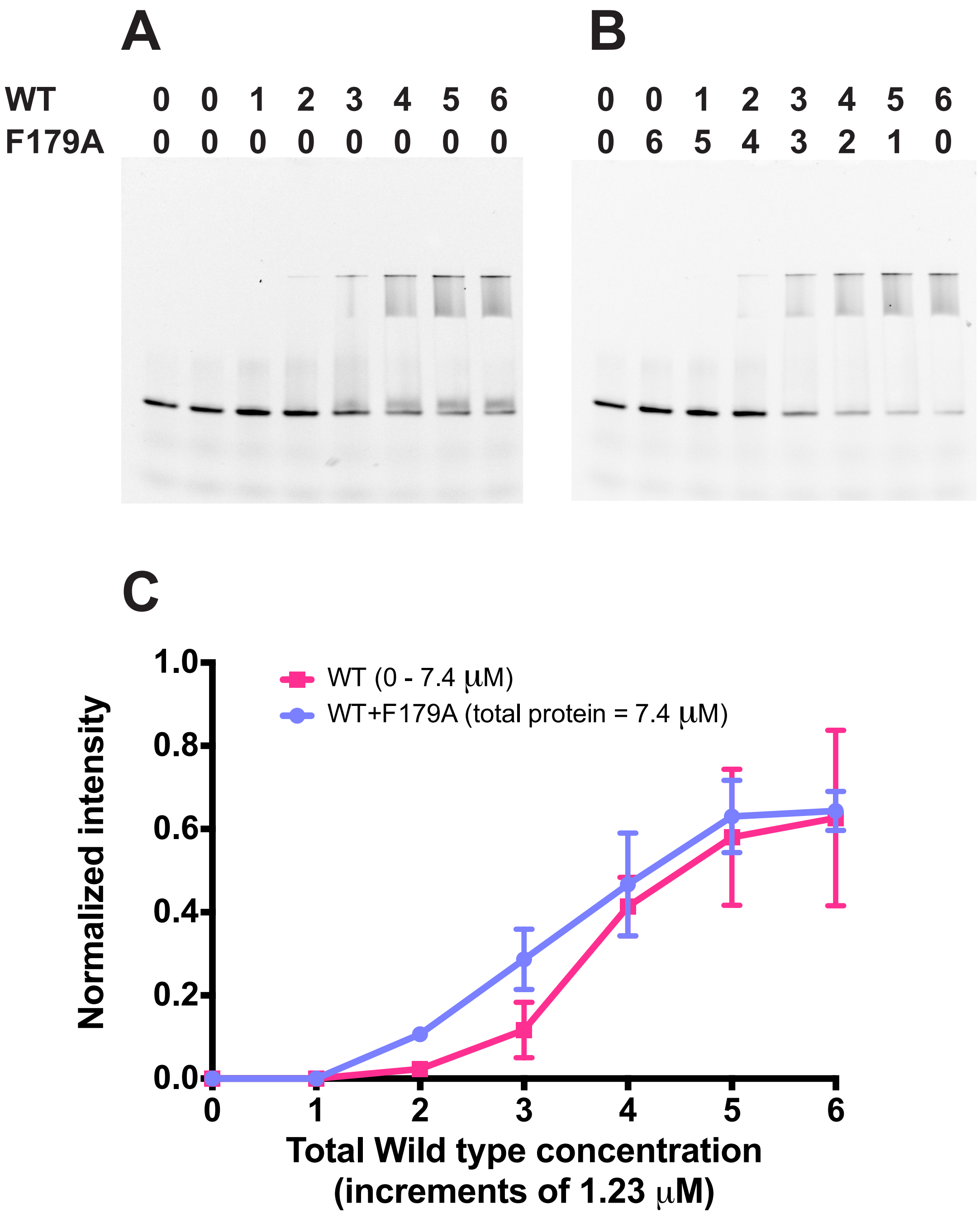

Supplementary Figure S10. Comparison of DNA-binding by *Pf*MCM$_N$-WT and *Pf*MCM$_N$-WT/*Pf*MCM$_N$-F179A mixtures. **A**) Representative EMSA titration of *Pf*MCM$_N$-WT in increments of 1.23 μM (0, 0, 1.23, 2.46, 3.69, 4.91, 6.14, 7.37 μM). **B**) Representative EMSA titration of *Pf*MCM$_N$-WT/*Pf*MCM$_N$-F179A in increments of 1.23 μM (*Pf*MCM$_N$-WT: 0, 0, 1.23, 2.46, 3.69, 4.91, 6.14, 7.37 μM; *Pf*MCM$_N$-F179A: 0, 7.37, 6.14, 4.91, 3.69, 2.46, 1.23, 0 μM). For this experiment, the total protein is constant at 7.37μM other than lane 1. Therefore, if *Pf*MCM$_N$-F179A were equivalent to wild-type, the level of binding would be uniform across the gel. Alternatively, if *Pf*MCM$_N$-F179A does not appreciably interact with wild-type or DNA, the outcome is expected to be very similar to panel A. **C**) Quantitation of the DNA-bound species for three independent trials of the two setups indicates very similar behavior. The level of DNA-binding is therefore most simply rationalized strictly by the overall concentration of *Pf*MCM$_N$-WT.

Supplementary Table S1. Analytical Ultracentrifugation Sedimentation Velocity

Sedimentation Velocity				
Sample	s_{20} (Svedberg)[a]	$s_{20,w}$ (Svedberg)[b]	*MW* (kDa)[c]	f/f_0[d]
**Apo-*Pf*MCM$_N$ (detecting interference)**				
*Pf*MCM$_N$-WT (76 µM)	2.49 (33%)	2.64	29.4	1.30
	4.08 (14%)	4.34	62.0	
	5.38 (13%)	5.72	93.7	
	7.08 (37%)	7.52	145.0	
*Pf*MCM$_N$-WT (180 µM)	2.30 (15%)	2.43	31.4	1.50
	4.35 (10%)	4.61	82.2	
	6.07 (15%)	6.44	135.5	
	7.35 (60%)	7.87	180.5	
*Pf*MCM$_N$-WT (350 µM)	6.97 (85%)	7.40	350.0	2.40
*Pf*MCM$_N$-ΔZFD (350 µM)	2.24 (95%)	2.37	28.0	1.35
*Pf*MCM$_N$-F179A (197 µM)	2.32 (99%)	2.46	30.0	1.42
*Pf*MCM$_N$-F179A (592 µM)	2.29 (97%)	2.43	30.9	1.46
*Pf*MCM$_N$-βT (65 µM)	2.10 (10%)	2.22	33.0	1.67
	5.78 (14%)	6.13	151.7	
	6.86 (68%)	7.27	196.1	
***Pf*MCM$_N$-WT + T40 (detecting interference)**				
*Pf*MCM$_N$-WT (20 µM)	2.57 (98%)	2.73	29.2	1.25
*Pf*MCM$_N$-WT + T40 (108 + 20 µM)	7.48 (82%)	7.94	240.0	1.80
*Pf*MCM$_N$-WT + T40 (38 + 7 µM)	7.73 (72%)	8.21	224.0	1.62
*Pf*MCM$_N$-WT + T40 (12.6 + 2.3 µM)	7.73 (55%)	8.20	200.0	1.50
***Pf*MCM$_N$ + Flc-T40 (detecting at 490nm)**				
Flc-T40 (20 µM)	1.73 (100%)	1.84	21.8 (22.1)*	1.52
*Pf*MCM$_N$-WT + Flc-T40 (54.6 + 10 µM)	1.67 (37%)	1.77	22.0 (22.1)	1.59
	7.77 (63%)	8.24	219.4	
*Pf*MCM$_N$-βT + Flc-T40 (92.8 + 17 µM)	1.66 (100%)	1.76	20.0 (22.1)	1.51
*Pf*MCM$_N$-F179A + Flc-T40 (125 + 23 µM)	1.68 (90%)	1.78	20.0 (22.1)	1.46
	2.70 (6%)	2.87	40.9	

[a] Total concentration based on protein fringe coefficient (84,326 fringes/(M.cm) and on Flc-T40 extinction coefficient 66,838 absorbance units/(M.cm).

[b] Sedimentation coefficient taken from the ordinate maximum of each peak in the best-fit *c(s)* distribution at 20°C with percentage signal amount in parenthesis.

[c] Standard sedimentation coefficient ($s_{20,w}$-value) in water at 20°C.

[d] Molar mass values (MW) taken from the *c(s)* distribution that was transformed to the *c(M)* distribution. The theoretical molar mass of the monomer is 29.315 kDa.

[c] Best-fit weight-average frictional ratio $(f/f_0)_w$ taken from the *c(s)* distribution.

[*] Apparent MW of DNA in parenthesis when partial specific volume is 0.73 mL/g (value of protein).

Supplementary Table S2. Analytical Ultracentrifugation Sedimentation Equilibrium

Sample	Model[a]	$K_{D1\text{-}X}$ *(µM)* [b] *(95% CI)*	K_{AB} *(µM)* [c] *(95% CI)*	*MW*[d] *(kDa)*	*RMSD*[e]
*Pf*MCM$_N$-WT (35 µM)	(6A↔[A]$_6$)	60 (48.0-74.0)			0.0029
*Pf*MCM$_N$-βT (12 µM)	(5A↔ [A]$_5$)	8.0 (7.4-8.4)			0.0018
**Protein+Flc-T40 490 nm**					
41.5 µM *Pf*MCM$_N$-WT + 7.6 µM Flc-T40	(B+[A]$_6$↔B[A]$_6$)		0.144 (0.143-0.146)		0.0019
19.1 µM *Pf*MCM$_N$-βT + 3.5 µM Flc-T40	Single species		No binding	21.5	0.0021
48.5 µM *Pf*MCM$_N$-F179A + 9.2 µM Flc-T40	Two discrete species		Not determined	52.1 (8%)	0.0029

Total loading concentrations of protein in *µM* and protein-DNA mixture, 83 and 15.2 µM respectively diluted.

[a] Reversible monomer-hexamer and monomer-pentamer self-association models as well as the reversible single site hetero-association model B+[A]$_6$↔B[A]$_6$ with B the monomer Flc-T40 species and A the hexamer *Pf*MCM$_N$–WT species [A]$_6$.

[b] Dissociation equilibrium constant at concentration where [monomer]=[hexamer] for *Pf*MCM$_N$-WT and [monomer]=[pentamer] for *Pf*MCM$_N$-βT. Errors represent the 95% CI using an automated surface projection method (4).

[c] Dissociation equilibrium constant for the reversible single-site heterogeneous association model B+[A]$_6$↔B[A]$_6$ with B the monomer Flc-T40 species and A the hexameric *Pf*MCM$_N$–WT treated as a single species in the analysis. Errors represent the 95% CI using an automated surface projection method (6).

[d] Single peak corresponds to adjusted MW of Flc-T40 with partial specific volume of 0.73 mL/g.

[e] Root mean square deviation of the fit, units in absorbance at 280 or 490 nm. Errors represent the 95% CI using an automated surface projection method (6).

Supplementary Table S3. Crystallographic Statistics

	*Pf*MCM$_N$-F179A	*Pf*MCM$_N$-βT	*Pf*MCM$_N$-ΔZFD
**Data collection**			
Space group	$P3_221$	$P3_221$	P1
Cell dimensions			
a, *b*, *c* (Å)	213.018, 213.018, 213.978	210.709, 210.709, 209.638	41.958, 50.964, 52.880
α, β, γ (°)	90, 90, 120	90, 90, 120	96.54, 94.77, 95.45
Resolution (Å)	50-3.20 (3.31-3.20)	50-3.20 (3.31-3.20)	50-1.55 (1.61-1.55)
R_{sym}	0.192 (1.000)	0.135 (0.640)	0.058 (0.082)
$I / \sigma I$	12.8 (2.6)	17.9 (3.9)	32.3 (13.0)
Completeness (%)	100.0 (100.0)	100.0 (100.0)	96.2 (94.5)
Redundancy	11.4 (11.5)	10.9 (10.1)	1.9 (1.9)
**Refinement**			
Resolution (Å)	50-3.20 (3.37-3.20)	50-3.20 (3.37-3.20)	50-1.55 (1.64-1.55)
No. reflections / Free	87902/4633 (12543/655)	82414/4328 (12037/636)	57024/3065 (8115/447)
R_{work} / R_{free}	0.202/0.230 (0.312/0.354)	0.237/0.270 (0.338/0.367)	0.217/0.250 (0.197/0.270)
No. atoms			
Protein	20310	20255	3034
ion	55	50	0
Water	0		203
B-factors			
Protein	66.663	69.870	19.237
ion	115.210	90.811	N/A
Water	N/A	N/A	24.637
R.m.s. deviations			
Bond lengths (Å)	0.007	0.012	0.010
Bond angles (°)	1.18	1.440	1.45

Supplementary References

1. Shima, N., Buske, T.R. and Schimenti, J.C. (2007) Genetic screen for chromosome instability in mice: Mcm4 and breast cancer. *Cell Cycle*, **6**, 1135-1140.

2. Shima, N., Alcaraz, A., Liachko, I., Buske, T.R., Andrews, C.A., Munroe, R.J., Hartford, S.A., Tye, B.K. and Schimenti, J.C. (2007) A viable allele of Mcm4 causes chromosome instability and mammary adenocarcinomas in mice. *Nat Genet*, **39**, 93-98.

3. Chuang, C.H., Yang, D., Bai, G., Freeland, A., Pruitt, S.C. and Schimenti, J.C. (2012) Post-transcriptional homeostasis and regulation of MCM2-7 in mammalian cells. *Nucleic Acids Res*, **40**, 4914-4924.

4. Fletcher, R.J., Bishop, B.E., Leon, R.P., Sclafani, R.A., Ogata, C.M. and Chen, X.S. (2003) The structure and function of MCM from archaeal M. Thermoautotrophicum. *Nat Struct Biol*, **10**, 160-167.

5. Froelich, C.A., Kang, S., Epling, L.B., Bell, S.P. and Enemark, E.J. (2014) A conserved MCM single-stranded DNA binding element is essential for replication initiation. *Elife*, **3**, e01993.

6. Zhao, H., Brautigam, C.A., Ghirlando, R. and Schuck, P. (2013) Overview of current methods in sedimentation velocity and sedimentation equilibrium analytical ultracentrifugation. *Curr Protoc Protein Sci*, **Chapter 20**, Unit20 12.
