# Supplementary material for: MCM ring hexamerization is a prerequisite for DNA-binding
Source: Nucleic Acids Res. 2015 Sep 13;43(19):9553–63. doi: 10.1093/nar/gkv914 (PMC4627082; doi:10.1093/nar/gkv914)
Supplement: SUPPLEMENTARY DATA [file supp_gkv914_nar-01263-m-2015-File008.docx]

# Supplementary Movie Legend

**Movie S1.** Crystal structure details for *Pf*MCM_N_-βT. The video is intended to illustrate the structural relationship between the *Pf*MCM_N_-WT hexamer (PDB: 4POF) and the *Pf*MCM_N_-βT pentamer, not to suggest actual molecular dynamics. The β-turn is projected into the central channel of the pentamer, similar to that of the hexamer. The asymmetric unit of the *Pf*MCM_N_-βT crystal structure contains a central pentameric ring with five peripheral monomers.
